# Systematic Review and Meta‐Analysis: Plant‐Based Therapy for Irritable Bowel Syndrome

**DOI:** 10.1002/ptr.70421

**Published:** 2026-08-09

**Authors:** Anselm Meyer, Jonas Auer, Lorena Cascant‐Ortolano, Niclas Schreiber, Amy Zimmermann‐Klemd, Carsten Gründemann, Philipp Busche, Jörg Schirra, Mark R. Fox

**Affiliations:** ^1^ Medical Department II University Hospital, Ludwig Maximilians‐University Munich Germany; ^2^ Clinic of Obstetrics and Gynaecology University Hospital Basel Basel Switzerland; ^3^ Departmental Library for the University Medical Center Johannes Gutenberg University Mainz Mainz Germany; ^4^ Department of Visceral, Vascular and Endocrine Surgery University Medical Center Halle (Saale), Martin‐Luther‐University Halle‐Wittenberg Halle (Saale) Germany; ^5^ Translational Complementary Medicine, Departement of Pharmaceutical Sciences University of Basel Basel Switzerland; ^6^ Laboratory and Clinic for Motility Disorders and Functional Digestive Diseases, Department of Integrative Gastroenterology Klinik Arlesheim Arlesheim Switzerland; ^7^ Division of Gastroenterology and Hepatology University Hospital Zurich Zurich Switzerland

**Keywords:** *Aloe vera*, dietary fiber, integrative medicine, irritable bowel syndrome, peppermint oil, systematic review and meta‐analysis, traditional Chinese herbal medicine

## Abstract

Irritable bowel syndrome (IBS) is a common disorder of brain‐gut interaction that presents with abdominal pain, bloating associated with variable stool consistency. Many IBS patients prefer plant‐based preparations to pharmacological therapy; however, the utility of this approach is not well established. To address this issue, a systematic review with meta‐analysis to assess the efficacy and safety of plant‐based therapy in IBS patients was performed. Literature search with 3 electronic databases (Medline via PubMed, Embase via Ovid, and Web of Science Core Collection) from inception to May 2024. Efficacy was assessed using dichotomous ratings of change in IBS symptoms. A random effects model with 95% confidence intervals was used. Through the screening process, 60 eligible studies were identified (6329 patients). Meta‐analysis was performed for fiber (8 studies, RR = 1.22; 95% CI: 1.00–1.49), peppermint oil (7 studies, RR = 1.63; 95% CI: 1.33–2.00), *Aloe vera* (3 studies, RR = 1.25; 95% CI: 0.76–2.06), and Chinese herbal medicine (7 studies, RR = 1.56; 95% CI: 1.27–1.92). For other plant‐based substances investigated in at least one randomized controlled trial (RCT) (e.g., St. John's wort, Anise oil), there were insufficient data for a meta‐analysis. The quality of many studies was limited. The findings demonstrate that the treatment of IBS patients with peppermint oil and Chinese herbal medicine were more effective than placebo and had a good safety profile. Dietary fiber showed effects that approached significance. Further, well‐powered RCTs are needed to establish the place of plant‐based preparations in IBS treatment.

**Abbreviations**
ADR: adverse drug reaction
CI: confidence interval
CO: crossover trial
IBS: irritable bowel syndrome
IBS‐C: IBS with constipation
IBS‐D: IBS with diarrhoea
NNT: number needed to treat
RCT: randomized controlled trial
RR: relative risk
TCM: Traditional Chinese herbal Medicine

## 1 Introduction

Irritable bowel syndrome (IBS) is characterised by a combination of abdominal pain, bloating and stool irregularity without evidence of serious disease (e.g., inflammation, neoplasia) on appropriate investigations (Lacy et al. 2016). The Rome IV criteria, introduced in 2016, classifies different IBS subtypes: with constipation, with diarrhoea, with mixed stool behaviour and not classified (Drossman and Hasler 2016). A recent meta‐analysis showed that the pooled prevalence worldwide is 3.8% (Oka et al. 2020), with women having a 46% higher incidence of IBS compared to men (more pronounced in western populations) (Oka et al. 2020). However, the prevalence of this disorder also depends on which diagnostic criteria are applied. For example, compared to Rome III, application of the Rome IV criteria has halved the apparent prevalence of IBS, as the previous version included abdominal discomfort in addition to pain in the definition (Palsson et al. 2020).

IBS is a disorder of gut‐brain interaction (previously termed “functional gastrointestinal disease”) (Black et al. 2020) that can have an important impact on quality of life (Gralnek et al. 2000). Both peripheral somatic and central nervous factors influence the development of IBS symptoms in a reciprocal or synergistic manner (Vanner et al. 2016). Abnormal physiology has been identified on multiple levels, including gastrointestinal motility, gut microbiome, mucosal and immune function, visceral sensitivity, and processing in the central nervous system (Drossman 2016). Further, social stress and psychological factors have been associated with the occurrence and severity of this condition (Van Oudenhove 2016). Accordingly, clinical assessment, endoscopy, and other routine investigations do not provide a positive diagnosis of IBS, and current guidelines do not provide a simple scheme for treatment (Layer 2021; Fox et al. 2018). This perceived lack of clarity can lead to repeated diagnostics and multiple treatments, with important direct and indirect costs for the individual, the health care system, and society (Tack et al. 2019; Elsenbruch and Benson 2021). It also leads to dissatisfaction with the “conventional” healthcare system (Houghton et al. 2016). As a result, at least 1 in 3 patients turn to complementary and alternative medicine (CAM) (Deutsch et al. 2020; Drossman et al. 2009). Plant‐based treatment, also known as “phytotherapy” or “herbal medicine,” is a key component of many CAM practices. This refers to the use of plants and plant‐derived substances to prevent, alleviate, or cure diseases either on its own or combined with conventional medical care (integrative medicine). Although widespread, the evidence base supporting the use of these preparations is variable, and the pharmacological mechanism of action is often both complex and uncertain (Fifi et al. 2018). Narrative reviews summarizing the use of plant‐based medicine in the management of IBS patients have been published; however, previous systematic reviews have generally investigated specific preparations or were published more than 15 years ago (Fifi et al. 2018; Liu et al. 2006; Shi et al. 2008; Alammar et al. 2019; Ingrosso et al. 2022; Zheng et al. 2021). This paper applied rigorous methodology to review the full range of plant‐based therapy available for this condition. This is relevant because (i) it is helpful to compare the relative efficacy of different preparations and (ii) more than one treatment may be required to manage the various symptoms of which IBS patients complain (e.g., both stool regulation *and* abdominal cramps) (Grundmann and Yoon 2014). The systematic review with meta‐analysis aims to provide a comprehensive and up‐to‐date assessment of the evidence base for plant‐based medications in the treatment of IBS patients that can act as a reference for primary care physicians and gastroenterologists.

## 2 Materials and Methods

The description of the methods is based on the PRISMA guidelines (Preferred Reporting Items for Systematic reviews and meta‐analyses) (Rethlefsen et al. 2021; Page et al. 2021). A protocol for this systematic review was published on Prospero (https://www.crd.york.ac.uk/prospero/display_record.php?ID=CRD42022322137).

### 2.1 Selection Criteria

Prospectively defined criteria for inclusion and exclusion were applied. Randomized controlled trials (RCT) that included standard therapy or placebo as comparators were included. Standard therapy for IBS differs between different geographical regions and centers and has changed over time; however, one can generalize that, in addition to diet and lifestyle recommendations, standard medical therapy for IBS includes preparations to regulate stool consistency and frequency (e.g., PEG, imodium) and also the use of spasmolytics to treat abdominal bloating and spasm (e.g., hyoscine butylbromide [Buscopan], pinaverium [Dicetel]) (Layer 2021; Fox et al. 2018). Additionally, other clinical trials without a control group that fulfilled the inclusion criteria were included in the systematic review, as long as more than 10 subjects participated. This decision was made because we wished to review the full range of plant‐based preparations that has been subjected to study.

For a study to be included, the diagnosis of IBS had to be established either by widely used criteria (e.g., Manning, Manning et al. 1978; Rome criteria, Mearin et al. 2016), or medical diagnosis of IBS. The latter was accepted because this diagnosis is often made in primary care without the use of formal criteria, and plant‐based preparations are often applied in this setting.

Clinical outcome was assessed in terms of improvement in IBS symptoms on the plant‐based intervention. Assessment tools included, for example, the IBS symptom severity score (Francis et al. 1997). Forced choice questions about “adequate relief of abdominal pain and discomfort” (Mangel et al. 1998) or reported change in symptoms such as abdominal pain or bowel movements were also accepted. As above, this decision was made to include as wide a range of studies as possible.

Exclusion criteria included homeopathic medicines (D3 or greater), unpublished or only partially published studies, patients under 18 years of age, studies with fewer than ten participants, and publications in languages other than German or English.

In case of lack of access to the full texts or insufficient information in the publications, the authors were contacted.

### 2.2 Search Strategy

A comprehensive literature search was initially run on 30th March 2022 and then rerun on 6th May 2024 in the following electronic databases: MEDLINE (PubMed), EMBASE (Ovid) and Web of Science Core Collection (Clarivate Analytics). Prior the execution of the search strategies developed by AM, these were peer reviewed. by an information specialist (LCO) using the PRESS (Peer Review of Electronic Search Strategies) checklist (Lefebvre and Duffy 2021). The search was limited to clinical trials of humans and no date or language limitations were applied. The reproducible search strategy for MEDLINE (PubMed) is available in Table 1.

**TABLE 1:** MEDLINE (PubMed) search strategy.

#1	“Diarrhea” [MeSH Terms:noexp] OR “Constipation” [MeSH Terms:noexp] OR “Irritable Bowel Syndrome” [MeSH Terms] OR “colonic diseases, functional” [MeSH Terms:noexp]
#2	“irritable bowel syndrom*” [Title/Abstract] OR “IBS‐D” [Title/Abstract] OR “IBS‐C” [Title/Abstract] OR “IBS” [Title/Abstract] OR “mucous colit*” [Title/Abstract] OR “colon irritable” [Title/Abstract] OR “diarrhea*” [Title/Abstract] OR “diarrhoea*” [Title/Abstract] OR “Constipation” [Title/Abstract] OR “Dyschezia” [Title/Abstract] OR “colonic inertia” [Title/Abstract] OR “functional colonic disease*” [Title/Abstract]
#3	#1 OR #2
#4	“medicine, traditional” [MeSH Terms:noexp] OR “Aromatherapy” [MeSH Terms] OR “Phytotherapy” [MeSH Terms:noexp] OR “plants, medicinal” [MeSH Terms]
#5	“pharmaceutical plant*” [Title/Abstract] OR “healing plant*” [Title/Abstract] OR “medicinal herb*” [Title/Abstract] OR “medicinal plant*” [Title/Abstract] OR “herbal medicine” [Title/Abstract] OR “medical plant*” [Title/Abstract] OR “aromatherap*” [Title/Abstract] OR “herb therap*” [Title/Abstract] OR “herbal therap*” [Title/Abstract] OR “phytotherap*” [Title/Abstract]
#6	“Plantago” [MeSH Terms] OR “Psyllium” [MeSH Terms]
#7	“Ispaghula” [Title/Abstract] OR “plantain*” [Title/Abstract] OR “plantago*” [Title/Abstract]
#8	“Mentha”[MeSH Terms]
#9	“spearmint*” [Title/Abstract] OR “mint*” [Title/Abstract] OR “mentha*” [Title/Abstract] OR “peppermint*” [Title/Abstract]
#10	“iberogast” [Supplementary Concept]
#11	“STW5” [Title/Abstract] OR “STW‐5” [Title/Abstract] OR “Iberogast” [Title/Abstract]
#12	“Aloe” [MeSH Terms]
#13	“aloe” [Title/Abstract]
#14	“Matricaria” [MeSH Terms]
#15	“Camomile” [Title/Abstract] OR “Chamomile” [Title/Abstract] OR “matricaria*” [Title/Abstract]
#16	“Kalanchoe” [MeSH Terms]
#17	“bryophyllum” [Title/Abstract] OR “kalanchoe” [Title/Abstract]
#18	“Flax” [MeSH Terms]
#19	“flax*” [Title/Abstract] OR “linum*” [Title/Abstract] OR “linseed*” [Title/Abstract]
#20	“Senna Plant” [MeSH Terms]
#21	“Cassia” [Title/Abstract] OR “Senna” [Title/Abstract]
#22	“Charcoal” [MeSH Terms]
#23	“Coal” [MeSH Terms] OR “Charcoal” [Title/Abstract] OR “coal*” [Title/Abstract]
#24	#4 OR #5 OR #6 OR #7 OR #8 OR #9 OR #10 OR #11 OR #12 OR #13 OR #14 OR #15 OR #16 OR #17 OR #18 OR #19 OR #20 OR #21 OR #22 OR #23 OR #24
#25	#3 AND #25
#26	“Animals” [Mesh] NOT (“Animals” [Mesh] AND “Humans” [Mesh])
#27	#25 NOT #26
#28	Therapy/broad [Filter]
#29	#27 AND #28

After additional studies were identified through automatic alerts, which were set up in the searched database and by looking at the citation of our included papers and relevant systematic reviews (citation chasing).

Search results were deduplicated using the Bramer method in EndNote (Bramer et al. 2016; Bramer and Bain 2017).

### 2.3 Screening

The citations were screened by AM and NS independently at the level of title and abstract. The full texts were then further screened for their suitability to be included in this systematic review. Both steps were performed in Covidence, a software application designed for systematic reviews that allows multiple reviewers to work independently of one another and in a blinded manner without knowledge of the other's decisions. At the full text screening stage, a reason was given for each study if it was excluded. In case of disagreement, consensus was sought through discussion, and the results were cross‐checked by the senior author MF.

### 2.4 Data Extraction

Data were summarized in tables according to the following aspects: General information: Author and year of publication, country in which the study was conducted, study design and treatment duration. Patient population: total number of study participants, drop‐out, age, sex, IBS definition and IBS subgroup. Study characteristics: plant‐based preparations, dosage, control, endpoints, adverse effects and outcome. A data extraction template was created in Covidence which AM and NS completed independently, and the results were cross‐checked by the senior author MF. Disagreements were resolved by consensus.

### 2.5 Risk of Bias Assessment

The Risk of Bias 2 (RoB 2) tool was applied as advised by the Cochrane Collaboration (Sterne et al. 2019). The following domains were assessed for each study: Bias risk from the randomization process, from deviations from the intended interventions (effect of allocation to the intervention), from missing outcome data, from outcome measurement, and from selection of the reported outcome. This checklist was completed by AM and NS independently in Excel and the result transferred to Covidence. Discrepancies were resolved by consensus.

### 2.6 Meta‐Analysis

A meta‐analysis was conducted for each preparation. The effectiveness rates in the intervention group compared to those in the comparator group were expressed as relative risk. This was recorded dichotomously as improvement or remission of IBS symptoms (thresholds defined in the original publications). A random effects model with 95% confidence intervals was applied (DerSimonian and Laird 1986). If RR > 1.0, then the therapy was more effective than placebo. If the confidence interval did not include 1.0, the intervention was significantly different to placebo. The number needed to treat with 95% confidence interval was calculated (NNT = 1 / (assumed control risk × [1—RR])).

Heterogeneity between studies was assessed with the Chi‐square (*χ* ^2^)‐test. A *p*‐value < 0.10 was used to define significant heterogeneity. The *I* ^2^ statistic was also used to test for heterogeneity among the included studies. A value between 0% and 24% did not indicate heterogeneity; 25%–49%, 50%–75%, and > 75% represented low, medium, and high heterogeneity, respectively (Higgins et al. 2003).

Forest plots were generated using Review manager, version 5.4.1 (The Cochrane Collaboration 2020). Funnel plots were to be assessed for signs of asymmetry. However, more than 10 studies had to be available for this (Sterne et al. 2011). In addition, a possible publication bias was to be determined using Eggers' test (Egger et al. 1997).

## 3 Results

### 3.1 Study Selection

The first literature search in the PubMed, Web of Science and Embase databases yielded 7894 citations. After removing duplicates, 6535 citations were screened at title and abstract level. Another five studies were found via a freehand search of citations in published systematic reviews and individual studies. As full texts, 117 studies were screened for eligibility. Of these, 56 studies could be included.

The update search found 8312 citations. In addition, two studies were found via email alerts. After deduplication, 3796 citations were screened. The second screening process resulted in the inclusion of four further studies. A summary of the review process is shown in the PRISMA flowchart (Figure 1).

**FIGURE 1:**
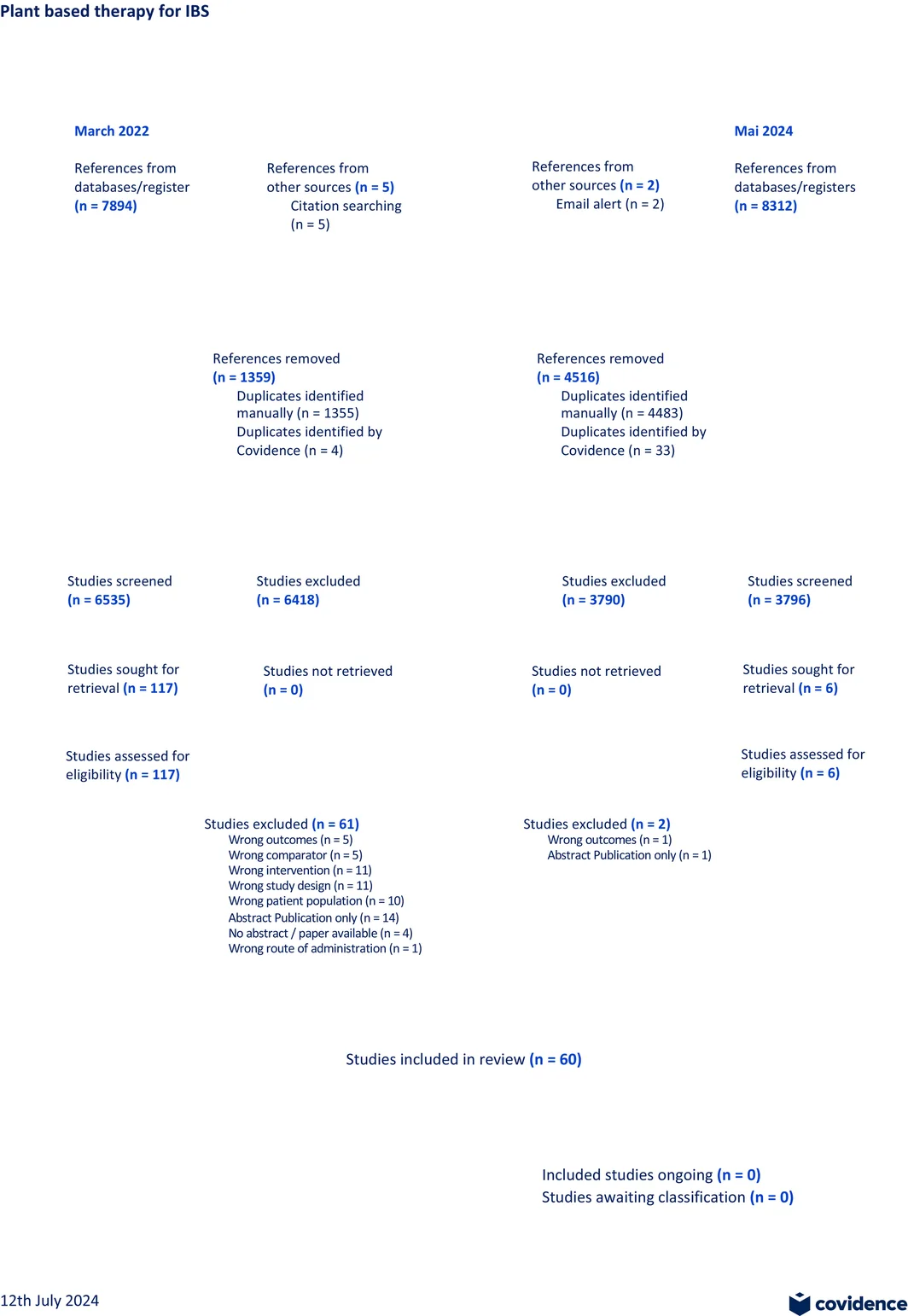
PRISMA flow diagram for study selection.

### 3.2 Study Characteristics

Sixty studies published between 1979 and 2024 reported results on a total of 6329 patients (11–1044 study participants). Where indicated, average age was between 27 and 50 years, and 3852 female and 2059 male patients were studied. Publications came from the following countries: 11 United Kingdom (Lucey et al. 1987; Rees et al. 2005, 1979; Snook and Shepherd 1994; Prior and Whorwell 1987; Cockerell et al. 2012; Fowlie et al. 1992; Davis et al. 2006; Hutchings et al. 2011; Cooke et al. 2000), 11 Iran (Merat et al. 2010; Agah et al. 2015; Bordbar et al. 2020; Derakhshande et al. 2019; Heydari et al. 2023; Jamalizadeh et al. 2022; Kazemian et al. 2017; Mosaffa‐Jahromi et al. 2016; Pazhouh et al. 2020; Vejdani et al. 2006; Hatami et al. 2020), 6 United States of America (Cash et al. 2016; Nee et al. 2021; van Tilburg et al. 2014; Brown et al. 2015; Mangel and Chaturvedi 2008; Saito et al. 2010), 5 China (Chen et al. 2018; Fan, Zheng, Lai, et al. 2017; Tang et al. 2018; Wang et al. 2006; Leung et al. 2006), 4 Germany (Lauche et al. 2016; Madisch, Holtmann, Plein, and Hotz 2004; Alt et al. 2017; Brinkhaus et al. 2005), 4 Australia (Lawson et al. 1988; Bensoussan et al. 2015, 1998; Hawrelak and Myers 2010), 3 Italy (Cappello et al. 2007; Bortolotti and Porta 2011; Portincasa et al. 2016), 2 Sweden (Ahluwalia et al. 2020; Størsrud et al. 2015), 2 the Netherlands (Bijkerk et al. 2009; Weerts et al. 2020), 2 Taiwan (Chang et al. 2010; Liu et al. 1997), 2 Iraq (Al‐Jassim 2019; Sahib 2013), 2 India (Jalihal and Kurian 1990; Suhasini Dehury et al. 2022), with 1 in Mexico (Remes‐Troche et al. 2021), Finland (Tarpila et al. 2004), Ireland (Arthurs and Fielding 1983), Japan (Matsueda et al. 2024), Austria (Hübner and Moser 2002), Israel (Sallon et al. 2002) and Bangladesh (Alam et al. 2013).

Various plant‐based preparations were studied including dietary fiber (12 studies; psyllium (Prior and Whorwell 1987; Jalihal and Kurian 1990; Arthurs and Fielding 1983), bran (Lucey et al. 1987; Rees et al. 2005; Snook and Shepherd 1994), linseeds (Cockerell et al. 2012; Tarpila et al. 2004) and combined fibers (Fowlie et al. 1992; Bijkerk et al. 2009; Chang et al. 2010; Remes‐Troche et al. 2021)), peppermint oil (11 studies (Rees et al. 1979; Merat et al. 2010; Mosaffa‐Jahromi et al. 2016; Cash et al. 2016; Nee et al. 2021; Lawson et al. 1988; Cappello et al. 2007; Weerts et al. 2020; Liu et al. 1997; Suhasini Dehury et al. 2022; Matsueda et al. 2024; Alam et al. 2013)), Traditional Chinese Herbal Medicine (TCM; 7 studies; Chen et al. 2018; Tang et al. 2018; Wang et al. 2006; Leung et al. 2006; Bensoussan et al. 2015; Bensoussan et al. 1998; Fan, Zheng, Lai, et al. 2017), *Aloe vera* (4 studies; Davis et al. 2006; Hutchings et al. 2011; Ahluwalia et al. 2020; Størsrud et al. 2015), and ginger (2 studies; van Tilburg et al. 2014; Al‐Jassim 2019). The other 24 studies investigated different plant‐based preparations (Cooke et al. 2000; Agah et al. 2015; Bordbar et al. 2020; Derakhshande et al. 2019; Heydari et al. 2023; Jamalizadeh et al. 2022; Kazemian et al. 2017; Mosaffa‐Jahromi et al. 2016; Pazhouh et al. 2020; Vejdani et al. 2006; Hatami et al. 2020; Brown et al. 2015; Mangel and Chaturvedi 2008; Saito et al. 2010; Lauche et al. 2016; Madisch, Holtmann, Plein, and Hotz 2004; Alt et al. 2017; Brinkhaus et al. 2005; Hawrelak and Myers 2010; Bortolotti and Porta 2011; Portincasa et al. 2016; Sahib 2013; Hübner and Moser 2002; Sallon et al. 2002).

Study designs included 49 RCTs (Rees et al. 2005; Prior and Whorwell 1987; Cockerell et al. 2012; Fowlie et al. 1992; Davis et al. 2006; Merat et al. 2010; Bordbar et al. 2020; Derakhshande et al. 2019; Heydari et al. 2023; Jamalizadeh et al. 2022; Kazemian et al. 2017; Mosaffa‐Jahromi et al. 2016; Pazhouh et al. 2020; Vejdani et al. 2006; Hatami et al. 2020; Cash et al. 2016; Nee et al. 2021; van Tilburg et al. 2014; Brown et al. 2015; Mangel and Chaturvedi 2008; Saito et al. 2010; Chen et al. 2018; Fan, Zheng, Lai, et al. 2017; Tang et al. 2018; Wang et al. 2006; Leung et al. 2006; Madisch, Holtmann, Plein, and Hotz 2004; Alt et al. 2017; Brinkhaus et al. 2005; Bensoussan et al. 2015, 1998; Cappello et al. 2007; Bortolotti and Porta 2011; Portincasa et al. 2016; Ahluwalia et al. 2020; Størsrud et al. 2015; Bijkerk et al. 2009; Weerts et al. 2020; Chang et al. 2010; Liu et al. 1997; Al‐Jassim 2019; Sahib 2013; Suhasini Dehury et al. 2022; Remes‐Troche et al. 2021; Tarpila et al. 2004; Arthurs and Fielding 1983; Hübner and Moser 2002; Sallon et al. 2002; Alam et al. 2013), 7 randomized controlled crossover trials (Lucey et al. 1987; Snook and Shepherd 1994; Rees et al. 1979; Hutchings et al. 2011; Lauche et al. 2016; Lawson et al. 1988; Jalihal and Kurian 1990) and 4 studies that compared the symptoms of IBS pre and post treatment (Cooke et al. 2000; Agah et al. 2015; Hawrelak and Myers 2010; Matsueda et al. 2024). Study duration ranged from 1 week to 5 months (most often 4 weeks [*n* = 24]).

### 3.3 Effect of Plant‐Based Preparations on IBS Symptoms

Overall, 34 of 60 (57%) studies reported a “positive” result (Rees et al. 1979; Cooke et al. 2000; Merat et al. 2010; Agah et al. 2015; Bordbar et al. 2020; Heydari et al. 2023; Jamalizadeh et al. 2022; Mosaffa‐Jahromi et al. 2016; Pazhouh et al. 2020; Vejdani et al. 2006; Hatami et al. 2020; Cash et al. 2016; Brown et al. 2015; Chen et al. 2018; Fan, Zheng, Lai, et al. 2017; Tang et al. 2018; Wang et al. 2006; Madisch, Holtmann, Plein, and Hotz 2004; Alt et al. 2017; Bensoussan et al. 1998; Cappello et al. 2007; Bortolotti and Porta 2011; Portincasa et al. 2016; Liu et al. 1997; Suhasini Dehury et al. 2022; Matsueda et al. 2024; Sallon et al. 2002; Alam et al. 2013). In 38 trials, the medicine in the intervention group was from a single plant (Lucey et al. 1987; Rees et al. 2005, 1979; Snook and Shepherd 1994; Prior and Whorwell 1987; Cockerell et al. 2012; Fowlie et al. 1992; Davis et al. 2006; Hutchings et al. 2011; Cooke et al. 2000; Merat et al. 2010; Agah et al. 2015; Derakhshande et al. 2019; Heydari et al. 2023; Mosaffa‐Jahromi et al. 2016; Cash et al. 2016; Nee et al. 2021; van Tilburg et al. 2014; Mangel and Chaturvedi 2008; Saito et al. 2010; Brinkhaus et al. 2005; Lawson et al. 1988; Cappello et al. 2007; Bortolotti and Porta 2011; Ahluwalia et al. 2020; Størsrud et al. 2015; Bijkerk et al. 2009; Weerts et al. 2020; Chang et al. 2010; Liu et al. 1997; Al‐Jassim 2019; Jalihal and Kurian 1990; Suhasini Dehury et al. 2022; Remes‐Troche et al. 2021; Tarpila et al. 2004; Arthurs and Fielding 1983; Matsueda et al. 2024; Alam et al. 2013). Whereas 22 trials investigated the effect of “herbal mixtures” on IBS symptoms (Bordbar et al. 2020; Jamalizadeh et al. 2022; Kazemian et al. 2017; Pazhouh et al. 2020; Vejdani et al. 2006; Hatami et al. 2020; Brown et al. 2015; Chen et al. 2018; Tang et al. 2018; Wang et al. 2006; Leung et al. 2006; Lauche et al. 2016; Madisch, Holtmann, Plein, and Hotz 2004; Alt et al. 2017; Bensoussan et al. 2015, 1998; Hawrelak and Myers 2010; Portincasa et al. 2016; Sahib 2013; Hübner and Moser 2002; Sallon et al. 2002; Fan, Zheng, Lai, et al. 2017).

Plant‐based therapy in IBS‐C was the focus of 8 studies. The use of flaxseed (Tarpila et al. 2004), kiwi fruit (Chang et al. 2010), and plant‐based anti‐IBS therapies from Tibet (Padma lax; Sallon et al. 2002), South America (Brown et al. 2015) or Iraq (Pazhouh et al. 2020) was shown to be helpful. Additionally, an extract of 7 selected ingredients from TCM (Bensoussan et al. 2015) relieved symptoms in IBS‐C patients. No overall benefit on IBS‐C symptoms was reported in studies on bran (Rees et al. 2005), non‐specific dietary fiber (Fowlie et al. 1992) or ginger (Al‐Jassim 2019).

Patients with IBS‐D were the focus of 9 studies. “Tong‐xie” and “Chang'an I” TCM preparations were both reported to provide superior symptom relief compared to placebo (Chen et al. 2018; Tang et al. 2018; Wang et al. 2006; Fan, Zheng, Lai, et al. 2017). In addition, peppermint oil (Alam et al. 2013), arrowroot (Cooke et al. 2000), and Dracocephalum kotschyi (dragonhead) (Heydari et al. 2023) were reported to be helpful. No improvement was shown in the studies using *Croton lechleri* (crofelmer) (Mangel and Chaturvedi 2008), an Ayurvedic herbal preparation (Lauche et al. 2016) and another TCM preparation (Leung et al. 2006).

### 3.4 IBS Definition and Study Inclusion

The Rome criteria were used frequently to include IBS patients Rome I (4; Rees et al. 2005; Bensoussan et al. 1998; Hübner and Moser 2002; Sallon et al. 2002), Rome II (13; Davis et al. 2006; Hutchings et al. 2011; Merat et al. 2010; Vejdani et al. 2006; Mangel and Chaturvedi 2008; Saito et al. 2010; Wang et al. 2006; Leung et al. 2006; Hawrelak and Myers 2010; Cappello et al. 2007; Bortolotti and Porta 2011; Alam et al. 2013; Bijkerk et al. 2004), Rome III (23; Cockerell et al. 2012; Agah et al. 2015; Bordbar et al. 2020; Derakhshande et al. 2019; Heydari et al. 2023; Kazemian et al. 2017; Mosaffa‐Jahromi et al. 2016; Cash et al. 2016; van Tilburg et al. 2014; Brown et al. 2015; Chen et al. 2018; Tang et al. 2018; Lauche et al. 2016; Alt et al. 2017; Bensoussan et al. 2015; Portincasa et al. 2016; Ahluwalia et al. 2020; Størsrud et al. 2015; Chang et al. 2010; Al‐Jassim 2019; Sahib 2013; Matsueda et al. 2024; Fan, Zheng, Lai, et al. 2017), and Rome IV (7; Jamalizadeh et al. 2022; Pazhouh et al. 2020; Hatami et al. 2020; Nee et al. 2021; Weerts et al. 2020; Suhasini Dehury et al. 2022; Remes‐Troche et al. 2021). The Krius‐ (Snook and Shepherd 1994; Jalihal and Kurian 1990) and Manning (Lucey et al. 1987; Cooke et al. 2000) criteria were both applied twice, and the Fielding criteria in 1 study (Arthurs and Fielding 1983). Another IBS definition provided the basis for inclusion in 7 studies (Prior and Whorwell 1987; Fowlie et al. 1992; Madisch, Holtmann, Plein, and Hotz 2004; Brinkhaus et al. 2005; Lawson et al. 1988; Liu et al. 1997; Tarpila et al. 2004). Further data regarding the characteristics of included studies, such as endpoints and results, are summarised in Tables 2 and 3.

**TABLE 2:** Characteristics of included studies.

Author year, country	Study‐design and (duration [weeks])	Completed/sample size (% female)	Mean age (years)	Diagnostic‐criteria (%Subtype)	Therapy vs. Control (dose)	Endpoints	Outcomes	Side effects (Intervention‐ vs. control group)
Fibre								
Bran (insoluble fibre)								
Lucey 1987, UK	CO (12 weeks)	28/44 (66%)	32 years	Manning	Bran biscuits vs. placebo (gradually increased until 15.6 g/days)	Total symptoms score	Individual symptom scores and TSS did not differ significantly between the bran group and the placebo group after three months therapy.	
Rees 2005, UK	RCT (8–12 weeks)	22/28 (86%)	36 years	Rome I (100% IBS‐C)	Coarse wheat bran vs. placebo (10–20 g/days)	Change in symptoms of abdominal pain, distention or flatulence	Overall feeling of improvement did not differ between groups.	
Snook 1994, UK	CO (7 weeks)	71/80 (84%)	40 years	Kruis (18% IBS‐C, 35% IBS‐D, 31% IBS‐M)	Coarse wheat bran vs. placebo (12 g/days)	Overall assessment of IBS‐symptoms	Individual symptoms of irritable bowel syndrome did not improve in the bran group compared to placebo group.	4 vs. 4 cases of intolerance
Ispaghula (soluble fibre)								
Arthurs 1983, Ireland	RCT (4 weeks)	78/80 (78%)	28 years	Fielding	Ispaghula husk vs. placebo (2 sachets/days)	Global improvement of IBS‐symptom	The response to ispaghula/poloxamer did not differ significantly to placebo.	
Bijkerk 2009, Netherlands	RCT (12 weeks)	164/275 (78%)	34 years	Rome II (56% IBS‐C, 25% IBS‐D, 19% IBS‐M)	Ispaghula (*n* = 85), bran (*n* = 97) vs. placebo (*n* = 93) (10 g)	Adequate relief of IBS related abdominal pain or discomfort	Severity of symptoms reduced: “…by 90 points, compared with 49 points in the placebo group (*p* = 0.03) and 58 points in the bran group (*p* = 0.61 versus placebo).”	Psyllium group: 63/85 Bran group: 62/97 Placebo group: 61/93 (e.g., worsening of symptoms)
Jalihal 1990, India	CO (4 weeks)	14/20 (20%)	38 years	Kruis (25% IBS‐C, 75% IBS‐D)	Ispaghula husk vs. placebo (30 g)	Global improvement of IBS‐ symptom	Global improvement of IBS‐symptoms occured significantly more in the husk group than in the placebo group (*p* = 0.05).	5 vs. 7 cases (e.g., worsening of symptoms)
Prior 1987, UK	RCT (12 weeks)	57/80 (90%)	Range: 18–63 years	Author‐defined IBS (49% IBS‐C, 9% IBS‐D, 42% IBS‐M)	Ispaghula husk vs. placebo (change in dose depending on stool consistency)	Overall improvement in wellbeing	Overall well‐being improved significantly in the ispaghula group compared to placebo group.	3 vs. 1 cases (e.g., nausea)
Linseeds (soluble fibre)								
Cockerell 2012, UK	RCT (4 weeks)	31/40 (66%)	42 years	Rome III (35% IBS‐C, 39% IBS‐D, 6% IBS‐M)	Whole (*n* = 14) or ground linseeds (*n* = 13) vs. placebo (*n* = 13) (2 spoons/days)	IBS severity scoring system	“Improvement in symptom severity did not reach statistical significance for whole linseeds (*n* = 11) versus ground linseeds (*n* = 11; *p* = 0.62), whole linseeds versus controls (*n* = 9; *p* = 0.12) and ground linseeds versus controls (*p* = 0.10).”	Whole linseeds group: 2 cases (e.g., metallic aftertaste)
Tarpila 2004, Finland	RCT (investigator blinded trial) followed by an open phase (12 weeks)	52/55 (91%)	45 years	Author‐defined IBS (100% IBS‐C)	Linseeds (*n* = 26) vs. Psyllium (*n* = 29) (6–24 g/days)	Change in constipation, abdominal bloating and discomfort	Constipation and abdominal symptoms decreased significantly in the linseed group but not in the psyllium group (*p* = 0.002).	
Other fibers								
Chang 2010, Taiwan	Pseudo‐randomised trial with placebo and healthy cohort as control (4 weeks)	70/76 (93%)	27 years	Rome III (100% IBS‐C)	Kiwifruit vs. placebo (2/days)	Frequency of bowel movements, stool volume, colonic transit time, Abdominal pain post defecation	Feeling uncomfortable or having abdominal pain after defecation was reported by significantly more patients in the IBS/CK group than in the other two groups (*p* < 0.05).	
Fowlie 1992, UK	RCT (12 weeks)	42/49 (65%)	40 years	Author‐defined IBS (100% IBS‐C)	Fibre (unspecified) vs. placebo	Global symptom score	Feeling ‘generally better’ did not differ between fibre group and placebo group.	
Remes‐Troche 2020, Mexico	RCT (1 week)	60/60 (78%)	35 years	Rome IV (47% IBS‐C, 20% IBS‐D, 33% IBS‐M)	Nopal fiber vs. placebo (4 g/days)	Adequate relief of IBS	Adequate relief of symptoms was reported by significantly more patients with nopal fiber 20 g/day (87%, *p* = 0.008) and 30 g/day (80%, *p* = 0.025) than with placebo (33%).	Nopal fiber group: 16 cases (e.g., diarrhoea)
Peppermint oil								
Alam 2013, Bangladesh	RCT (6 weeks)	65/74 (14%)	29 years	Rome II (100% IBS‐D)	Peppermint oil vs. placebo (3 × 2 ml/days)	Changes in IBS‐symptoms and quality of life	Abdominal pain improved significantly in the peppermint oil group compared to the placebo group (*p* > 0.001).	
Cappello 2007, Italy	RCT (4 weeks)	50/57 (76%)	41 years	Rome II	Peppermint oil vs. placebo (2 × 225 mg/days)	IBS symptoms score	In the mean total IBS symptoms score a ≥ 50% reduction occurred significantly more in the peppermint oil group than in the placebo group.	Peppermint oil group: 1 case (heartburn)
Cash 2016, USA	RCT (4 weeks)	70/72 (75%)	41 years	Rome III (53% IBS‐D, 47% IBS‐M)	Peppermint oil vs. placebo (3 × 180 mg/days)	Total IBS Symptom Score	TISS decreased by 40% with peppermint oil compared compared to 24.3% with placebo (*p* = 0.0246).	
Dehury 2022, India	RCT (4 weeks)	60/60 (58%)	36 years	Rome IV (15% IBS‐C, 33% IBS‐D, 52% IBS‐M)	Peppermint oil vs. placebo (3 × 180 mg/days)	Total IBS Symptom Score Numeric Pain Rating Scale	TISS and PAIN score decreased significantly with peppermint oil compared to placebo at 24 h and 4 weeks.	
Lawson 1988, Australia	CO (4 weeks)	18/25 (84%)	50 years	Author‐defined IBS	Peppermint oil vs. placebo (3 capsules/days)	Change in IBS‐Symptoms	Global severity score did not change significantly.	Peppermint oil group: 10 cases (e.g., perianal burning)
Liu 1997, Taiwan	RCT (4 weeks)	101/110 (40%)	Range: 18–70 years	Author‐defined IBS	Peppermint oil vs. placebo (3–4 capsules/days)	Change in IBS‐Symptoms	Symptom improvements were significantly better with Colpermin than with placebo (*P* < 0.05).	Peppermint oil group: 2 cases (e.g., heartburn)
Matsueda 2024, Japan	Multi‐center, non‐controlled, open‐label trial (4 weeks)	67/69 (46%)	38 years	Rome III (26% IBS‐C, 51% IBS‐D, 23% IBS‐M)	Peppermint oil (3 × 187 mg [0.2 mL]/days)	Patient's global assessment IBS symptom severity score	Patient's global assessment improvement rate was 85.1%; the IBS symptom severity score was significantly lowered.	Peppermint oil Group: 14 cases (e.g., hypersensitivety)
Merat 2010, Iran	RCT (8 weeks)	60/90 (75%)	36 years	Rome II	Peppermint oil vs. placebo (3 capsules/days)	Change in IBS‐Symptoms and quality of life	Abdominal pain was reduced significantly in the Colpermin group compared to controls.	19 vs. 14 cases (e.g., heartburn)
Nee 2021, USA	RCT (6 weeks)	101/133 (74%)	43 years	Rome IV (20% IBS‐C, 41% IBS‐D, 35% IBS‐M)	Peppermint oil vs. placebo (3 × 180 mg/days)	IBS‐Symptom Severity Score	Improvement was numerically lower in the peppermint oil group than in the placebo group, but the difference was not statistically significant (*p* = 0.97).	27 vs. 22 cases (e.g., heartburn)
Rees 1979, UK	CO (3 weeks)	16/18			Peppermint oil vs. placebo (2 × 1‐2 capsules/days)	Change in IBS‐Symptoms	In the overall assessment patients felt significantlybetter in the peppermint oil group compared with the placebo group (*P* < 0–01).	Peppermint oil group: 2 cases (heartburn)
Weerts 2020, Netherlands	RCT (8 weeks)	178/189 (78%)	34 years	Rome IV (22% IBS‐C, 44% IBS‐D, 21% IBS‐M)	Peppermint oil vs. placebo (182 mg)	Abdominal pain response, overall relief of IBS‐Symptoms	“Abdominal pain response did not differ significantly between peppermint oil and placebo groups”	Peppermint oil group: 368/125 Placebo group: 100/64 (e.g., heartburn and belching)
*Aloe vera*								
Ahluwalia 2020, Sweden	RCT (4 weeks)	160/173 (80%)	45 years	Rome III (30% IBS‐C, 26% IBS‐D, 44% IBS‐M or undefined)	*Aloe vera* vs. placebo (2 × 250 mg/days)	IBS‐Symptom Severity Score	“Overall severity of symptoms reduced in both Aloe and control treatment groups (*P* < 0.001), without difference between groups (*P* = 0.62).”	2 vs. 5 cases (e.g., worsening of nausea, bloating, and gas)
Davis 2006, UK	RCT (4 weeks)	41/58 (78%)	Range: 18–65 years	Rome II (21% IBS‐C, 61% IBS‐D, 18% IBS‐M)	*Aloe vera* vs. placebo (4 × 50 ml/days)	Global summated symptom score	Global summated symptom score did not differ between groups (*p* = 0.763).	2 vs. 4 cases (e.g., vomiting and nausea)
Hutchings 2011, UK	CO (20 weeks)	47/110 (76%)	47 years	Rome II	*Aloe vera* vs. placebo (2 × 60 ml/days)	Quality of Life	IBS quality of life did not differ between *Aloe vera* and placebo groups.	
Størsrud 2015, Sweden	RCT (4 weeks)	63/68 (75%)	45 years	Rome III (7% IBS‐C, 29% IBS‐D, 63% IBS‐M)	*Aloe vera* vs. placebo (2 effervescent tablets/days)	IBS Symptom Severity Score	Overall severity of symptoms decreased in the *Aloe vera* group but not in the placebo group, with no difference between groups (*p* = 0.10).	Placebo group: 1 case (worsening of nausea)
Chinese herbal medicine								
Bensoussan 2015, Australia	RCT (8 weeks)	108/125 (93%)	49 years	Rome III (100% IBS‐C)	Chinese Herbal Medicine formulation vs. placebo (2 × 5 capsules/days)	Adequate relief of IBS, IBS Symptom Severity Score	Adequate relief was reported more often in the CHM group compared to the placebo group (*p* = 0.010); but the formulation was ineffective for abdominal pain and flatulence.	33 vs. 29 cases (e.g., loose stools and associated bloating/cramping)
Bensoussan 1998, Australia	RCT (16 weeks)	99/116 (65%)	47 years	Rome I	Individualized Chinese herbal formulation (*n* = 38), standard Chinese herbal formulation (*n* = 43) vs. placebo (*n* = 35) (3 × 5 capsules/days)	Total bowel symptom score, global improvement of IBS‐ symptom	Bowel symptom score improved significantly in both standard and individualized CHM groups compared with the placebo group (*P* = 0.03).	1 vs. 1 case (e.g., upper gastrointestinal discomfort)
Chen 2018, China	RCT (4 weeks)	155/160 (55%)	34 years	Rome III (100% IBS‐D)	Tong‐Xie‐Yao‐Fang (TXFY) vs. placebo (2×/d)	Adequate relief of IBS‐D	Adequate relief of global symptoms had a significantly higher rate in the TXFY group compared to the placebo group (*p* = 0.017).	3 vs. 3 cases (e.g., nausea)
Fan 2017, China	RCT (4 weeks)	780/1044 (57%)	36 years	Rome III (100% IBS‐D)	Tongxie vs. placebo (3×/days)	Change in abdominal pain	All 6 symptoms assessed decreased significantly in the tongxie group, compared to the placebo group (*P* < 0.001).	30 vs. 12 cases (e.g., headache and nausea)
Leung 2006, China	RCT (8 weeks)	95/119 (52%)	45 years	Rome II (100% IBS‐D)	Chinese Herbal Medicine formulation vs. placebo (2 × 1 package dissolved in hot water/days)	Improvement of global IBS symptoms	Global symptom improvement did not differ between TCM and placebo groups.	2 vs. 1. cases (e.g., skin rash)
Tang 2018, China	RCT (8 weeks)	196/216 (38%)	43 years	Rome III (100% IBS‐D)	Chang'an I Recipe vs. placebo (3 × 1 bag/days)	IBS‐Symptom Severity Score, adequate relief of IBS	Total effective rate was 67.6% in the treatment group and 40.2% in the control group.	
Wang 2006, China	RCT (3 weeks)	51/60 (52%)	37 years	Rome II (100% IBS‐D)	Tong‐xie‐ning (TXNG) vs. placebo (3 × 5 g/days)	IBS‐related “effects of pain on daily life”	IBS‐related pain reduction was reported by 82.7% in the TXNG group compared with 39.3% in the placebo group (*p* = 0.001).	
Ginger								
Al‐Jassim 2019, Iraq	RCT (20 days)	45/45	18 years and over	Rome III (100% IBS‐C)	Ginger (1 g/d) vs. placebo vs. Brewer's yeast (500 mg/days)	IBS severity scale visual analog scale for IBS	Abdominal pain reduction in the ginger group was not significant (*p* < 0.05).	
van Tilburg 2014, USA	RCT (4 weeks)	44/45	18 years and over	Rome III (100% IBS‐D)	Ginger (1 or 2 g/days) vs. placebo	IBS severity scale, adequate relief of symptoms scale	Number of treatment responders did not differ between 1 g ginger, 2 g ginger and placebo groups (*p* > 0.05).	5 vs. 5 cases (e.g., heartburn and nausea)

*Note:* Study design: Crossover trial (CO), randomized controlled trial (RCT).

**TABLE 3:** Characteristics of included studies.

Author Year, Country	Study‐design and (duration [weeks])	Completed/sample size (% female)	MeanAge (Years)	Diagnostic‐criteria (%Subtype)	Therapy vs. Control (Dose)	Endpoints	Outcomes	Side effects (intervention‐ vs. control group)
Other plant‐based preparations								
Agah 2015, Iran	Pre‐ vs. post‐ treatment studie (4 weeks)	45/45	Range: 10–50 years	Rome III	Chamomile (2 × 10 drops/days)	Change in IBS symptoms	IBS symptoms were reduced at the second and fourth week (*p* < 0.001).	
Alt 2017, Germany	RCT (8 weeks)	86/90 (70%)	46 years	Rome III	Xanthofen (IQP‐CL‐101) vs. placebo (2 × 330 mg/days)	IBS‐Symtom Severity Score	IBS‐SSS decreased more strongly with IQP‐CL‐101 than placebo after 8 weeks (−113 vs. −39 points; *p* < 0.001).	
Bordbar 2020, Iran	RCT (2 weeks)	58/64 (61%)	34 years	Rome III (23% IBS‐C, 36% IBS‐D, 41% IBS‐M)	Herbal Softgel Capsule (essential oils: Zataria multiflora Boiss, *Trachyspermum ammi* L. and *Anethum graveolens* L.) vs. hyoscine butylbromi‐de (2 × 180 mg/days)	IBS Symptom Severity Scale, IBS Adequate Relief, IBS Global Assessment Improvement	Posttreatment improvement percentage for IBS‐AR, IBS‐GAI, and IBS‐SSS was higher in the intervention group (83.9%, 75%, and 87%) than in the control group (37.9%, 27.5%, and 34.4%), (*p* < 0:001).”	2 vs. 10 cases (e.g., abdominal pain)
Bortolotti 2011, Italy	RCT (6 weeks)	42/50 (40%)	45 years	Rome II	Red pepper vs. placebo (4 × 150 mg/d, in 8 patients reduction to 2 × 150 mg/days)	Abdominal pain and bloating	Abdominal pain and bloating mean score values were significantly improved in the red pepper group but not in the placebo group.	Red pepper group: 14 cases (abdominal pain)
Brinkhaus 2005, Germany	RCT (18 weeks)	90/106 (63%)	48 years	Author‐defined IBS	Curcuma xanthorriza (*n* = 24) *Fumaria officinalis* (*n* = 24) vs. placebo (*n* = 58) (3 × 20 mg/days Curcuma xanthorriza 3 × 250 mg/days *Fumaria officinalis* )	IBS‐related pain and distension	No therapeutic benefit for fumitory or curcuma over placebo.	1 (Curcuma group) vs. 8 (Fumaria group) vs. 1 (Placebo group) cases (e.g., constipation and distension)
Brown 2015, USA	RCT (2 weeks)	13/16 (81%)	38 years	Rome III (100% IBS‐C)	Quebracho, Conker Tree and Mentha balsamea Willd oil vs. placebo (150 mg Quebracho, 470 mg Conker Tree and 0.2 ml M. balsamea Wild oil)	Change in constipation and bloating	The average constipation (*p* = 0.0034), bloating (*p* < 0.001) and constipation plus bloating scores (*p* < 0.001) significantly improvent in the herbal extracts group but not in the placebo group.	
Cooke 2000, UK	pre‐ vs. post‐treatment studie (4 weeks)	10/11 (45%)	39 years	Manning (100% IBS‐D)	Arrowroot (3 × 10 ml/days)	Improvement in IBS symptoms	Abdominal pain (z = 2.3, *p* = 0.02) and diarrhoea (z = 2.0, *p* = 0.05) improved during treatment.	Arrowroot group: 6 cases (e.g., constipation)
Derakhshande 2019, Iran	RCT (4 weeks)	84/90 (63%)	34 years	Rome III (34% IBS‐C, 30% IBS‐D, 29% IBS‐M)	Achillea wilhelmsii C. Koch vs. placebo (2 × 250 mg/days)	IBS‐symptom severity index, IBS‐Quality of life	IBS‐Quality of life improved in the Achillea wilhelmsii C. Koch group and in the placebo group, with no significant difference between groups (*p* > 0.05).	
Hatami 2020, Iran	RCT (4 weeks)	80/112 (58%)	37 years	Rome IV (21% IBS‐C, 16% IBS‐D, 63% IBS‐M)	Cumin Sofouf vs. Mebeverine (3 × 3 g sachets/days)	IBS‐Symptom Severity Score	“Cumin sofouf was significantly more effective than mebeverine.”	12 vs. 3 cases (e.g., headache and vomiting)
Hawrelak 2010, Australia	two arm, open‐label, uncontrol‐led pilot study (3 weeks)	29/31 (84%)	41 years	Rome II (32% IBS‐C, 68% IBS‐D or IBS‐M)	IBS‐D formulation and IBS‐C formulation: (2 × 1 scoop powder dissolved in 250 mL apple juice/days)	Bowel movements/day Consistency and frequency of stool Abdominal pain severity	Bowel habit improved with the C‐IBS formula but not with the DA‐IBS formula in IBS‐D or ‐M. A number of IBS symptoms improved with both formulas.	IBS‐D group: 2 cases (e.g., constipation)
Heydari 2023, Iran	RCT (4 weeks)	76/101 (55%)	37 years	Rome III (100% IBS‐D)	Dracocephalum kotschyi Boiss. vs. placebo (3 × 75 mg/days)	IBS‐Severity scoring system IBS‐Quality of life. Tempera‐ment Checklist	Quality of life, temperament, and IBS symptoms improved during treatment.	
Hübner 2002, Austria, Belgium, Israel and Morocco	RCT (12 weeks)	144/262 (67%)	42 years	Rome I	Activated charchoal (Eucarbon) vs. non‐activated charcoal (carbo ligni) (1–3, 4–6, > 6 tablets/days)	overall well‐being	Symptom improvement with Eucarbon was 60%, with a 9% higher efficacy compared to carbo ligni.	7 vs. 5 cases (affecting the gastrointestinal tract)
Jamalizadeh 2021, Iran	RCT (4 weeks)	134/150 (64%)	37 years	Rome IV (41% IBS‐C, 16% IBS‐D, 34% IBS‐M)	Zataria multiflora Boiss and *Trachyspermum copticum* (L.) capsules vs. mebeverine or placebo (2/days)	Improvement in IBS symptoms	Pain, bloating, and reflux decreased significantly more in the ZT group than in the placebo and mebeverine groups after 4 weeks (*p* < 0.05).	
Kazemian 2017, Iran	RCT (4 weeks)	42/60 (36%)	39 years	Rome III (23% IBS‐C, 6% IBS‐D, 70% IBS‐M)	Boswellia carterii, *Zingiber officinale* , and *Achillea millefolium* vs. placebo (3 capsules/days)	IBS‐Severity scoring system IBS‐Quality of life	Abdominal pain, bloating, depression and anxiety decreased in the herbal medication group, but not statistically significant.	Boswellia carterii, *Zingiber officinale* , and *Achillea millefolium* group: 4 cases (e.g., diarrhea)
Lauche 2016, Germany	CO (4 weeks)	21/32 (59%)	50 years	Rome III (100% IBS‐D)	Ayurvedic herbs vs. placebo (2 × 5 g powder dissolved in 100 mL water/days)	IBS‐symptom severity score	IBS symptom intensity did not differ between the Ayurvedic herbs group and the placebo group (difference 24.10; 95% CI: −17.12; 65.32, *p* = 0.26).	9 vs. 4 cases (e.g., diarrhoea, stomach cramps and skin rash)
Madisch 2004, Germany	RCT (4 weeks)	203/208 (60%)	44 years	Author‐defined IBS (31% IBS‐C, 27% IBS‐D, 41% IBS‐M)	STW 5 (*n* = 51), STW 5 II (*n* = 52) and bitter candytuft mono‐ extract (*n* = 53) vs. placebo (*n* = 52) (3 × 20 drops/days)	IBS symptoms score Abdominal pain scale	Abdominal pain score and irritable bowel syndrome symptom score were significantly reduced in the STW 5 and STW 5‐II group compared to placebo group after 4 weeks.	1 (STW 5 group) vs. 1 (bitter candytuft group) case (e.g., constipation)
Mangel 2008, USA	RCT (12 weeks)	49–67/245 (74%)	50 years	Rome II (100% IBS‐D)	Crofelemer (125 (*n* = 62), 250 (*n* = 59) or 500 mg [*n* = 62]) vs. placebo	Stool consistency adequate relief	Stool consistency or adequate relief were not significantly improved in the Crofelemer group compared to the placebo group.	58 vs. 21cases (e.g., worsening of symptoms)
Mosaffa‐Jahromi 2016, Iran	RCT (4 weeks)	108/120 (49%)	34 years	Rome III (33% IBS‐C, 36% IBS‐D, 18% IBS‐M)	Anise oil capsules (*n* = 40) or peppermint oil capsules (*n* = 40) vs. placebo (*n* = 40) (3/days)	Improvement in IBS symptoms IBS‐Quality of Life	IBS symptoms improved significantly more with AnisEncap than placebo or Colpermin after the 4‐weeks (…) (*p* < 0.0001).	0 (Anise oil group) vs. 10 (Peppermint oil group) vs. 1 (Placebo group) cases (e.g., heartburn)
Pazhouh 2020, Iran	RCT (6 weeks)	52/70 (71%)	34 years	Rome IV (100% IBS‐C)	Anti‐IBS Sirup vs. placebo (3 × 15 ml/days)	IBS Symptom Severity Scale. Bristol Stool Form Scale	IBS‐SSS and BSFS scores significantly decreased compared to baseline after 6‐week intervention in both groups (*p* < 0.001). Both scores in the treatment group were lower than in the placebo group after the intervention (*p* = 0.041).	Anti‐IBS Sirup group: 5 cases (e.g., headache)
Portincasa 2016, Italiy	RCT (4 weeks)	116/121 (66%)	40 years	Rome III (38% IBS‐C, 62% IBS‐D)	Capsules (curcumin and fennel essential oil) vs. placebo (2/days)	IBS‐ symptom severity score IBS‐quality of life	IBS‐SSS significantly decreased after 30 days of treatment (50.05% ± 28.85% vs. 26.12% ± 30.62%, *p* < 0.001).	
Sahib 2013, Iraq	RCT (8 weeks)	40	Range: 25–60 years	Rome III	Capsules ( *Mentha longifolia* , *Cyperus rotundus* and *Zingiber officinale* ) vs. mebeverine (3/days)	Change in IBS‐Symptoms	IBS symptoms improved with the herbal combination, withs results comparable to Mebeverine after 8 weeks of tretmeant.	
Saito 2010, USA	RCT (12 weeks)	60/70 (86%)	42 years	Rome II (30% IBS‐C, 39% IBS‐D, 31% IBS‐M)	St John's wort vs. Placebo (2 × 450 mg/days)	Self‐reported overall bowel symptom score	BSS decreased in the placebo group significantly lower than in the SJW group at 12 weeks (*p* = 0.03).	Placebo group: 1 case (heartburn)
Sallon 2002, Israel	RCT (12 weeks)	61/80 (73%)	47 years	Rome I (100% IBS‐C)	Padma Lax vs. placebo (2 capsules/days)	Effect on abdominal pain stool pattern	Constipation, severity of abdominal pain, and its effect on daily activities, incomplete evacuation, abdomi‐ nal distension and flatus/flatulence improved significantly in the Padma Lax group compared to placebo after 3 months.	10 vs. 5 cases (e.g., diarrhea)
Vejdani 2006, Iran	RCT (8 weeks)	28/32 (44%)	39 years	Rome II (38% IBS‐C, 31% IBS‐D, 31% IBS‐M)	Carmint plus Loperamide or psyllium vs. placebo (3 × 30 drops/days)	Abdominal pain overall relief of IBS symptoms	Severity and frequency of abdominal pain/discomfort were significantly lower in the Carmint group than the placebo group (*p* = 0.016, *p* = 0.001).	4 vs. 3 cases (e.g., malaise)

*Note:* Study design: Crossover trial (CO), randomized controlled trial (RCT).

### 3.5 Risk of Bias Within Studies

#### 3.5.1 Randomized Controlled Trials

A high risk of bias was present in 11 studies (Prior and Whorwell 1987; Merat et al. 2010; Jamalizadeh et al. 2022; Kazemian et al. 2017; Vejdani et al. 2006; Bortolotti and Porta 2011; Chang et al. 2010; Sahib 2013; Suhasini Dehury et al. 2022; Arthurs and Fielding 1983; Sallon et al. 2002), some concern of bias was present in 32 studies (Rees et al. 2005; Cockerell et al. 2012; Fowlie et al. 1992; Davis et al. 2006; Bordbar et al. 2020; Derakhshande et al. 2019; Heydari et al. 2023; Pazhouh et al. 2020; Hatami et al. 2020; Cash et al. 2016; van Tilburg et al. 2014; Brown et al. 2015; Mangel and Chaturvedi 2008; Fan, Zheng, Lai, et al. 2017; Tang et al. 2018; Wang et al. 2006; Leung et al. 2006; Madisch, Holtmann, Plein, and Hotz 2004; Alt et al. 2017; Brinkhaus et al. 2005; Bensoussan et al. 1998; Cappello et al. 2007; Portincasa et al. 2016; Ahluwalia et al. 2020; Størsrud et al. 2015; Bijkerk et al. 2009; Liu et al. 1997; Al‐Jassim 2019; Remes‐Troche et al. 2021; Tarpila et al. 2004; Hübner and Moser 2002; Alam et al. 2013) whereas 6 studies had a low risk of bias (Mosaffa‐Jahromi et al. 2016; Nee et al. 2021; Saito et al. 2010; Chen et al. 2018; Bensoussan et al. 2015; Weerts et al. 2020). The main reasons for concern included lack of clarity on the randomization process or allocation sequence concealment and/or failure to perform intention‐to‐treat analysis. In addition, most authors did not publish a protocol, so it was not possible to assess whether the endpoints were pre‐specified. It was noted that the risk of bias for outcome measurement in the RCTs was mainly rated as low. Figure 2 summarizes the risk of bias in the included studies.

**FIGURE 2:**
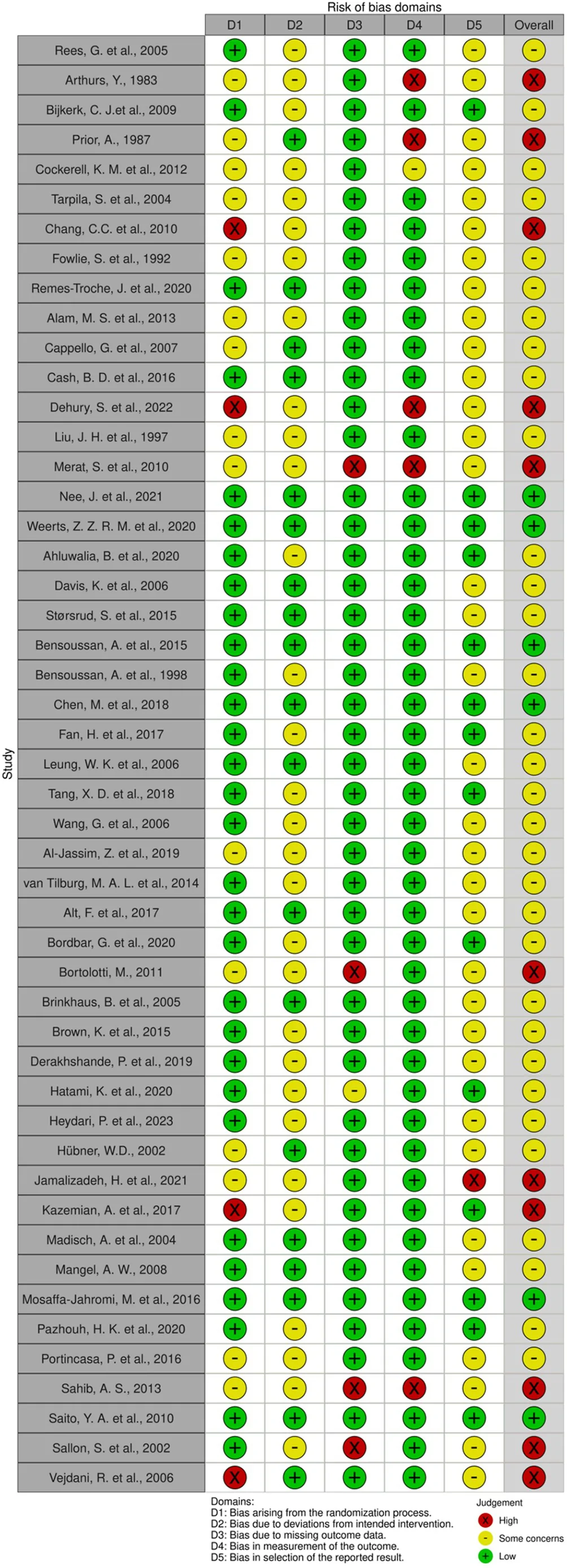
Risk of bias assessment of randomized controlled trials (McGuinness and Higgins 2020).

#### 3.5.2 Randomized Controlled Crossover Studies

All 7 randomized controlled crossover trials received the assessment “some concern” (Lucey et al. 1987; Snook and Shepherd 1994; Rees et al. 1979; Hutchings et al. 2011; Lauche et al. 2016; Lawson et al. 1988; Jalihal and Kurian 1990). This was mainly due to a lack of information on the randomization process, missing outcome data, and failure to perform an intention‐to‐treat analysis. In contrast, the risk of bias for the effect of transmission and that of outcome measurement was mainly rated as low. Figure 3 shows the result of the risk of bias survey.

**FIGURE 3:**
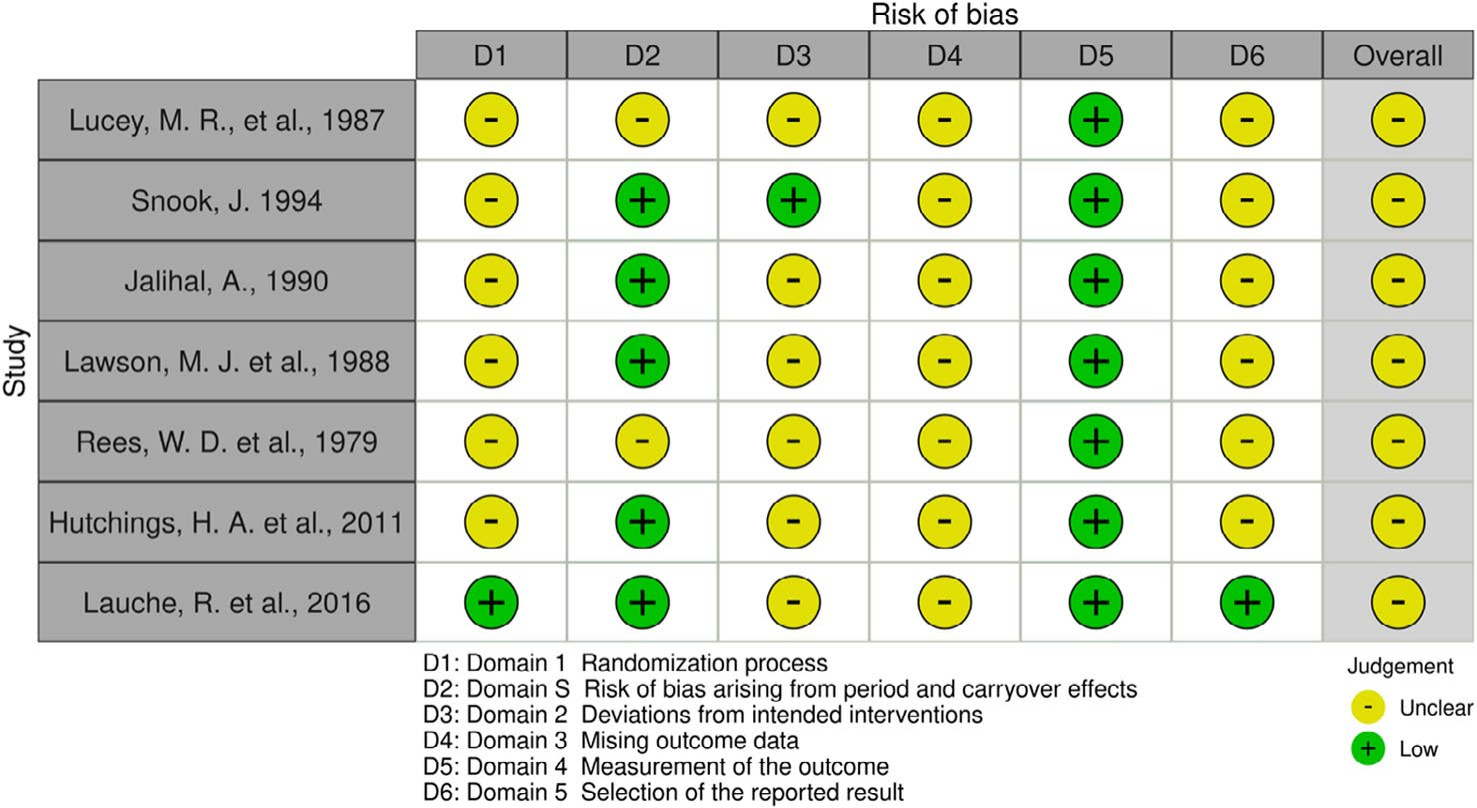
Risk of bias assessment of randomized controlled crossover studies (McGuinness and Higgins 2020).

### 3.6 Meta‐Analysis

#### 3.6.1 Meta‐Analysis for Dietary Fiber

Of the 12 studies that assessed the efficacy of dietary fiber (soluble and insoluble), 8 studies had sufficient data to include in a meta‐analysis that examined the effect on global IBS symptoms (Lucey et al. 1987; Rees et al. 2005; Prior and Whorwell 1987; Cockerell et al. 2012; Fowlie et al. 1992; Bijkerk et al. 2009; Remes‐Troche et al. 2021; Arthurs and Fielding 1983). Seven hundred thirty three patients made up the population for the intention‐to‐treat analysis. Fiber provided a benefit for 192 of 387 patients in the intervention group. In the control group, placebo intake was helpful for 127 of 346 patients (RR = 1.22; 95% CI: 1.00–1.49, Figure 4). There was low heterogeneity (*I* ^2^ = 33%, *p* = 0.16).

**FIGURE 4:**
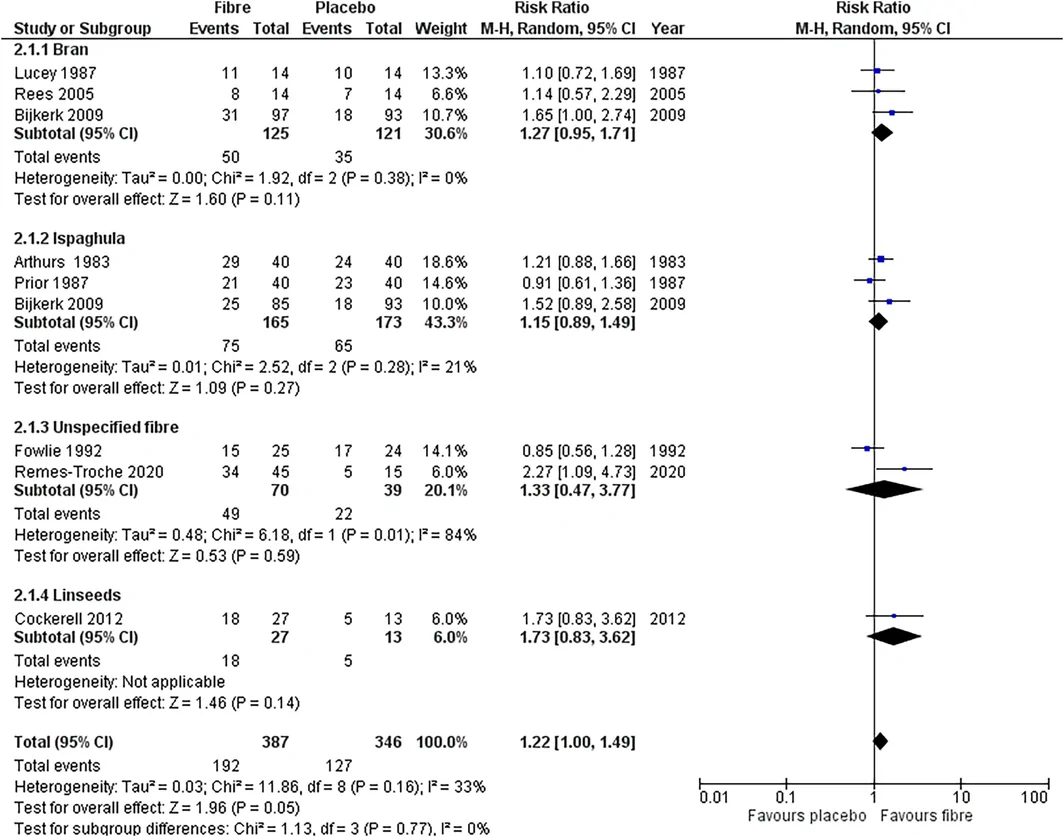
Forest plot of randomized controlled trials of dietary fiber.

Separate meta‐analyses were performed to assess the efficacy of soluble and insoluble fiber. In three studies on insoluble fiber (Bran), the intention‐to‐treat analysis consisted of 246 patients (Lucey et al. 1987; Rees et al. 2005; Bijkerk et al. 2009). Taking bran provided relief for 50 out of 125 patients. In the placebo group, there was symptom relief for 35 of 121 patients (RR = 1.27; 95% CI: 0.95–1.71). There was no heterogeneity for the meta‐analysis on bran (*I* ^2^ = 0%, *p* = 0.38). In studies with soluble fiber (e.g., psyllium, ispaghula), 338 patients completed the intention‐to‐treat analysis (Prior and Whorwell 1987; Bijkerk et al. 2009; Arthurs and Fielding 1983). The intervention was helpful for 75 of 165 patients. In the control, 65 of 173 patients reported an improvement (RR = 1.15; 95% CI: 0.89–1.49). The level of heterogeneity was low (*I* ^2^ = 21%, *p* = 0.28).

#### 3.6.2 Meta‐Analysis for Peppermint Oil

Of the 10 studies that investigated peppermint oil, 7 were selected for meta‐analysis. The primary endpoint of 5 studies was general symptom improvement of IBS (Mosaffa‐Jahromi et al. 2016; Cash et al. 2016; Nee et al. 2021; Cappello et al. 2007; Weerts et al. 2020) and relief of abdominal pain in 2 studies (Merat et al. 2010; Liu et al. 1997). The intention‐to‐treat analysis consisted of 668 patients. Of the 311 patients treated with peppermint oil, 144 reported benefits. The control group showed positive effects in 97 of 357 patients (RR = 1.63; 95% CI: 1.33–2.00, Figure 5). The NNT with peppermint oil was 5.84 (95% CI: 3.68–11.5). There was no heterogeneity in this meta‐analysis (*I* ^2^ = 0%, *p* = 0.58).

**FIGURE 5:**
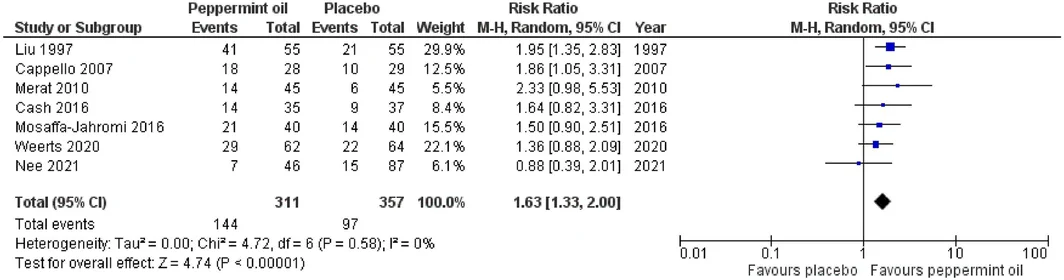
Forest plot of randomized controlled trials of peppermint oil.

#### 3.6.3 Meta‐Analysis for *Aloe vera*

Of the 4 studies on *Aloe vera* , 3 were included in a meta‐analysis (Davis et al. 2006; Ahluwalia et al. 2020; Størsrud et al. 2015). The intention‐to‐treat analysis included 299 patients. In the *Aloe vera* group, 62 of the 155 patients experienced an improvement in the general symptoms of IBS, whereas 51 of 144 patients in the control group reported positive effects (RR = 1.25; 95% CI: 0.76–2.06, Figure 6). Symptom relief was measured using the IBS Symptom Severity Score, with patients with a reduction of 50 out of a possible 500 points being considered responders. Heterogeneity for the meta‐analysis on *Aloe vera* was moderate (*I* ^2^ = 56%, *p* = 0.1).

**FIGURE 6:**
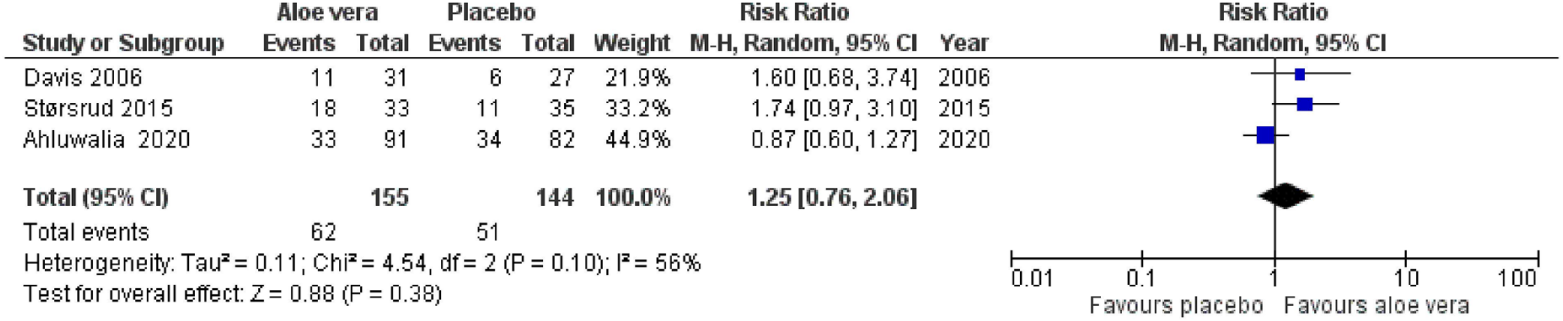
Forest plot of randomized controlled trials of *Aloe vera* .

#### 3.6.4 Meta‐Analysis for Traditional Chinese Herbal Medicine

All 7 studies on TCM herbal mixtures could be included in the meta‐analysis (Chen et al. 2018; Fan, Zheng, Lai, et al. 2017; Tang et al. 2018; Wang et al. 2006; Leung et al. 2006; Bensoussan et al. 2015, 1998). 1454 patients were included in the intention‐to‐treat analysis. In 6 studies, the primary outcome measure was relief of global symptoms of IBS (Chen et al. 2018; Tang et al. 2018; Wang et al. 2006; Leung et al. 2006; Bensoussan et al. 2015, 1998), and 1 study applied improvement in abdominal pain (Fan, Zheng, Lai, et al. 2017). Therapy with TCM herbal mixtures was successful in 403 of 731 patients. In the placebo group, 245 of 723 patients reported symptom improvement (RR = 1.56; 95% CI: 1.27–1.92, Figure 7). The NNT with TCM was 5.27 (95% CI: 3.21–10.93). The test for heterogeneity showed that there was moderate heterogeneity (*I* ^2^ = 60%, *p* = 0.02).

**FIGURE 7:**
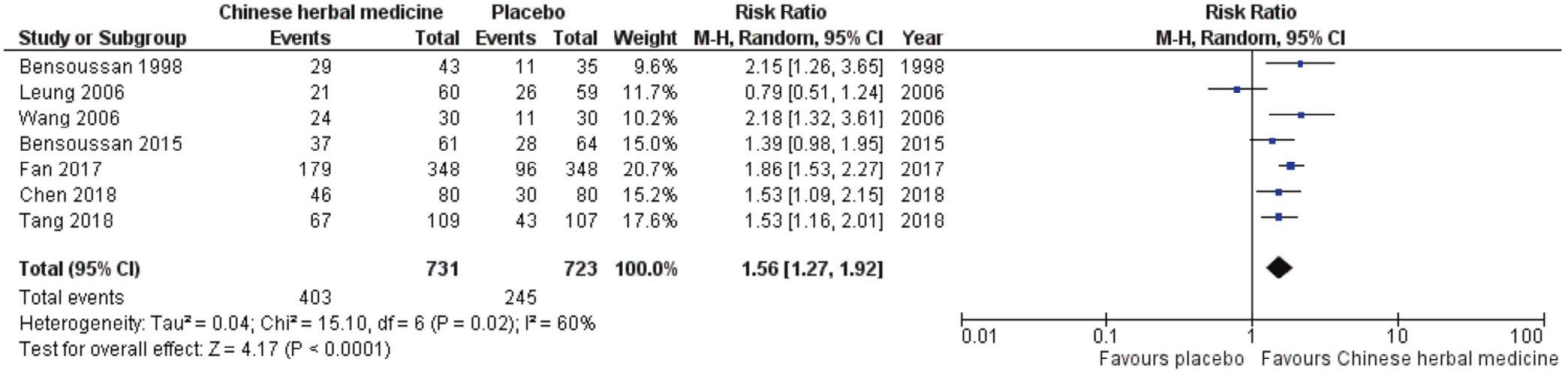
Forest plot of randomized controlled trials of Traditional Chinese herbal Medicine.

#### 3.6.5 Publication Bias

The number of studies in each meta‐analysis was too small to test for publication bias (at least 10 studies required).

### 3.7 Adverse Events With Plant‐Based Preparations in IBS Treatment

Overall, nearly two‐thirds of studies (39/60 [65%]; Snook and Shepherd 1994; Prior and Whorwell 1987; Cockerell et al. 2012; Rees et al. 1979; Davis et al. 2006; Cooke et al. 2000; Merat et al. 2010; Bordbar et al. 2020; Kazemian et al. 2017; Mosaffa‐Jahromi et al. 2016; Pazhouh et al. 2020; Vejdani et al. 2006; Hatami et al. 2020; Nee et al. 2021; van Tilburg et al. 2014; Mangel and Chaturvedi 2008; Saito et al. 2010; Chen et al. 2018; Fan, Zheng, Lai, et al. 2017; Leung et al. 2006; Lauche et al. 2016; Madisch, Holtmann, Plein, and Hotz 2004; Brinkhaus et al. 2005; Lawson et al. 1988; Bensoussan et al. 2015, 1998; Hawrelak and Myers 2010; Cappello et al. 2007; Bortolotti and Porta 2011; Ahluwalia et al. 2020; Størsrud et al. 2015; Bijkerk et al. 2009; Weerts et al. 2020; Liu et al. 1997; Jalihal and Kurian 1990; Remes‐Troche et al. 2021; Matsueda et al. 2024; Hübner and Moser 2002; Sallon et al. 2002) reported adverse drug reactions (ADRs). A total of 832 adverse events were reported from 2596 patients that received plant‐based preparations in the intervention groups with 328 adverse events in 1920 patients who received placebo. In terms of severity, ADRs ranged between mild and moderate.

#### 3.7.1 Dietary Fiber

ADRs were reported in 6/12 (50%) trials on fiber (Lucey et al. 1987; Rees et al. 2005; Snook and Shepherd 1994; Prior and Whorwell 1987; Cockerell et al. 2012; Fowlie et al. 1992; Bijkerk et al. 2009; Chang et al. 2010; Jalihal and Kurian 1990; Remes‐Troche et al. 2021; Tarpila et al. 2004; Arthurs and Fielding 1983), with 150 adverse events recorded in 385 patients taking the active preparations and 78 in 252 patients that received the control. Intake of psyllium led to 71 adverse events in 145 patients (placebo: 69/153) whereas intake of bran led to 65 reports in 177 patients (placebo: 66/173). Side effects were flatulence and diarrhoea.

#### 3.7.2 Peppermint Oil

ADRs were reported in 9/12 (75%) trials with peppermint oil (Rees et al. 1979; Merat et al. 2010; Mosaffa‐Jahromi et al. 2016; Cash et al. 2016; Nee et al. 2021; Lawson et al. 1988; Cappello et al. 2007; Weerts et al. 2020; Liu et al. 1997), with 460 adverse events reported by 485 patients in the intervention group and 137 in 313 patients in the control group. Heartburn, belching, reflux, and headache were among the adverse effects that occurred.

#### 3.7.3 *Aloe vera*

ADRs were reported in 3/4 studies with *Aloe vera* (Davis et al. 2006; Ahluwalia et al. 2020; Størsrud et al. 2015). Only 4 adverse effects were reported by 153 patients on the active preparation (including nausea and vomiting) with 9 events in 142 patients in the control groups.

#### 3.7.4 Traditional Chinese Herbal Medicine

ADRs were reported in 5/7 (71%) trials using herbal mixtures from TCM (Chen et al. 2018; Leung et al. 2006; Bensoussan et al. 2015, 1998; Fan, Zheng, Lai, et al. 2017), with 70 adverse events reported by 630 patients on active medication and 45 events by 586 patients receiving the control. Events such as diarrhoea, flatulence, dizziness, and nausea were reported.

#### 3.7.5 Ginger

One study on ginger reported adverse events (Al‐Jassim 2019), with 5 adverse events in 30 patients in the intervention group and 5 in 15 patients in the control group.

#### 3.7.6 Other Plant‐Based Preparations

Similar numbers of ADRs were reported in 16/24 (67%) trials reporting results in other plant‐based medicines (Cooke et al. 2000; Bordbar et al. 2020; Kazemian et al. 2017; Mosaffa‐Jahromi et al. 2016; Pazhouh et al. 2020; Vejdani et al. 2006; Hatami et al. 2020; Mangel and Chaturvedi 2008; Saito et al. 2010; Lauche et al. 2016; Madisch, Holtmann, Plein, and Hotz 2004; Brinkhaus et al. 2005; Hawrelak and Myers 2010; Bortolotti and Porta 2011; Hübner and Moser 2002; Sallon et al. 2002). Eight studies did not report adverse events (Agah et al. 2015; Derakhshande et al. 2019; Heydari et al. 2023; Jamalizadeh et al. 2022; Brown et al. 2015; Alt et al. 2017; Portincasa et al. 2016; Sahib 2013).

## 4 Discussion

This systematic review with meta‐analysis summarizes results from studies on plant‐based treatment of IBS. Searches of three independent databases identified 60 unique studies with a total of 6329 patients. Sufficient data was available to perform meta‐analysis for four interventions: fiber, peppermint oil, *Aloe vera* , and TCM. The results show that treatment with peppermint oil and TCM improve IBS symptoms compared to placebo, with borderline positive results for fiber. The safety of these plant‐based preparations was confirmed.

### 4.1 Dietary Fiber

The meta‐analysis suggests that dietary fiber can improve IBS symptoms compared to control interventions (RR = 1.22; 95% CI: 1.00–1.49); however, this difference did not reach statistical significance (confidence interval includes “1.0”) and the risk of bias assessment revealed concerns in several studies. Additionally, adverse drug reactions (ADRs) such as bloating and flatulence were often reported. Detailed analysis showed that this was more common after ingestion of non‐soluble fiber (e.g., bran) than soluble fiber (e.g., milled psyllium or flax seeds). These results are consistent with the findings of Bijkerk et al. that randomized patients to receive psyllium, bran or placebo (Bijkerk et al. 2009). It was shown that psyllium improved, whereas bran exacerbated, IBS symptoms and the number of dropouts in the bran group was twice as high as in the other two groups because of side effects (Bijkerk et al. 2009). In‐line with these findings, two previous meta‐analyses also demonstrated superior efficacy of soluble fiber over both bran and placebo in the treatment of this disorder (e.g., Nagarajan et al. RR = 1.49; 95% CI: 1.09–2.03) (Moayyedi et al. 2014; Nagarajan et al. 2015).

The action of different dietary fibers in the digestive tract may explain this discrepancy. Soluble fibers bind and hold water in the intestine, and this can improve stool consistency both in constipation and diarrhea (McRorie et al. 1998; Singh 2007). By contrast, bran does not bind water and acts by mechanical stimulation of the intestinal epithelium promoting peristalsis and secretion, effects that can be helpful in constipation (Tomlin and Read 1988; Lewis and Heaton 1999). Another difference is that bran, but not psyllium or other soluble fibers used in IBS treatment, is fermented by colonic bacteria. This produces gas and short‐chain fatty acids, which distend the intestine and increase salt and water secretion, respectively (Eswaran et al. 2013). In many IBS patients this can lead to abdominal cramps, bloating, and diarrhea, typical of “food intolerance” (Misselwitz et al. 2019).

Overall, the results support the strong recommendation made by expert guidelines that soluble dietary fibers, such as milled psyllium or flax seeds, can be prescribed to treat IBS patients, especially to control stool frequency and consistency (Lacy et al. 2021).

### 4.2 Peppermint Oil

The meta‐analysis shows that peppermint oil provides significant symptom relief in IBS patients compared to placebo (RR = 1.63; 95% CI: 1.33–2.00) with a number needed to treat of 5.84 (95% CI: 3.68–11.5). The risk of bias was limited or low in most studies. Notwithstanding this finding, the most recent study included in the analysis, a high‐quality paper by Weerts et al., did not show statistically significant effects (Weerts et al. 2020). The reasons for this may include: (i) the requirement to meet highly stringent endpoints to achieve primary outcomes, (ii) the comparison of placebo with two different galenic preparations that released peppermint oil either in the proximal small intestinal or in the ileo‐colonic region. Only the former had positive effects on abdominal pain in IBS patients, and this effect was smaller than that expected from the prospective power calculation used in study planning (Weerts et al. 2020).

Adverse drug reactions such as heartburn or burning discomfort in the upper abdomen were more common in the intervention than in the placebo group (e.g., Nee et al. 48% vs. 31%, respectively; Nee et al. 2021); however, the severity was generally mild.

These results are consistent with a previous meta‐analysis by Alammar et al. that reported significant benefit of peppermint oil compared to placebo both for the treatment of global symptoms of IBS (RR = 2.39; 95% CI: 1.93–2.97), and abdominal pain (RR = 1.78; 95% CI: 1.43–2.20) (Alammar et al. 2019). Similarly, Ingrosso et al. reported that the “relative risk of *not* improving global IBS symptoms” was 0.65 (95% CI: 0.47–0.88) (Ingrosso et al. 2022). The meta‐analyses differ in search strategies, include some different studies and, on occasion, differ in risk of bias assessment. Despite these differences, the positive findings in all three studies supports the conclusion that peppermint oil can provide significant benefit in IBS patients. Further a recent, positive study by Suhasini Dehury et al. could not be included in the current study because the primary outcome was presented as the relative change in Total IBS Symptom Score compared to baseline (Suhasini Dehury et al. 2022). For meta‐analysis, the exact number of patients in the intervention group and in the control group that meet the threshold for clinical improvement is required.

High dose peppermint oil, equivalent to more than 50 cups of peppermint tea in each tablet, has pharmacological effects (Grigoleit and Grigoleit 2005). Smooth muscle relaxation is thought to be mediated by menthol, which blocks calcium channels (Hawthorn et al. 1988). This may explain the reduction in abdominal cramps and pain; however, relaxation of the lower esophageal sphincter may also lead to reflux symptoms (Sigmund and McNally 1969). Menthol may also have direct, analgesic effects by inhibiting the nociceptors TRPM8 and TRPA1 (Chumpitazi et al. 2018; Liu et al. 2013; Karashima et al. 2007). Additionally, antibacterial properties of peppermint oil may reduce fermentation in the large bowel (Thompson et al. 2013).

In summary, there is good evidence that peppermint oil can relieve symptoms in IBS patients; however, not all high‐quality studies delivered positive findings. Given this heterogeneity, the strength of recommendation in published guidelines is low or moderate (Chang et al. 2022; Savarino et al. 2022).

### 4.3 *Aloe vera*

Superiority of *Aloe vera* over placebo was not shown in any of the 4 published studies included in this study, and a meta‐analysis of the pooled data provided no evidence that *Aloe vera* improves symptoms in IBS patients (RR = 1.25; 95% CI: 0.76–2.06).

This finding contrasts with a systematic review by Hong et al. (2018); however, this analysis includes a crossover study by Hutchings et al. (2011), that did not meet the entry criteria because data was summarized and not presented separately for the first treatment phase. Additionally, Hong et al. did not include the well‐powered randomized controlled study by Ahluwalia et al. that found no overall benefit of *Aloe vera* compared to placebo (Ahluwalia et al. 2020). A post hoc analysis of two studies by the same authors in IBS patients with diarrhea (Ahluwalia et al. 2020; Størsrud et al. 2015), suggests that *Aloe vera* could alleviate the severity of certain symptoms, especially abdominal pain, in this subgroup (Ahluwalia et al. 2021); however, this has not yet been confirmed.

Preclinical studies have shown that multiple components in *Aloe vera* (e.g., aloin, alin, acemannen) have potential mucosal protectant, analgesic and anti‐inflammatory effects that could be helpful in gastrointestinal disease (Sánchez et al. 2020; Ghosh et al. 2011; Hajhashemi et al. 2012; Langmead et al. 2004). It also contains fructans and other polysaccharides prebiotics that can promote the growth of Bifidobacterium spp. and other components of a healthy microbiome (Gullón et al. 2015; Quezada et al. 2017). Indeed, a relationship between the composition of the microbiome and the effect of therapy with *Aloe vera* has been reported (Ahluwalia et al. 2020). However, as discussed in the section on fiber, the fermentation of fructans in the large intestine can cause bloating and other abdominal symptoms. Such unwanted effects could explain why not more IBS patients respond to this preparation.

*Aloe vera* can be used in patients with irritable bowel syndrome. However, due to the current state of studies, when included in published guidelines, the strength of recommendation is very weak (Layer 2021).

### 4.4 Traditional Chinese Herbal Medicine (TCM)

“Tongxie” preparations are commonly used in TCM for the treatment of digestive disorders. Pooled data in IBS patients show a significant positive effect compared to control preparations (RR = 1.56; 95% CI: 1.27–1.92), with a number needed to treat of 5.27(95% CI: 3.21–10.93). Adverse reactions were no more frequent in the intervention than the control groups and were never severe. The risk of bias assessment identified some concerns in most studies, related to lack of allocation sequence concealment (i.e., blinding) or lack of transparency related to the protocol. Additionally, although four plant based products are found in most of the TCM preparations tested (see below), the investigational products are not identical.

A previous systematic review by Zheng et al. compared the effect of “Chinese herbal mixtures” and control preparations in 10 trials in IBS patients (Zheng et al. 2021). This meta‐analysis shows that TCM herbal mixtures improve the “general symptoms of IBS” (RR = 1.76; 95% CI: 1.33–2.33), and abdominal pain (RR = 1.85; 95% CI: 1.59–2.14). Three trials were not included in the current analysis because of the use of individualized rather than standardized preparations (Wang et al. 2018), the lack of a true control arm (Su et al. 2013) or other exclusion criteria (Shih et al. 2019). Another meta‐analysis by Liang et al. summarized the effect of “Tongxie Yaofang” in IBS patients with diarrhea (Liang et al. 2022). Again, a significant effect compared with placebo was identified (RR = 1.12; 95% CI: 1.02–1.22); however, the authors concluded that no clear recommendation can be made. More studies have been published in Chinese literature; however, review of the English abstracts identified exclusion criteria, and these were not included in the systematic review.

Tongxie was first described in the Yuan Dynasty (1271–1368) and consists of four basic substances: Paeoniae Radix Alba (White Peony), Saposhnikoviae Radix (Laserwort), Citri Reticulatae Pericarpium (Mandarin), and Atractylodis Macrocephalae (“Cang Zhú”). Three studies included in the meta‐analysis contained these substances in a standardized mixture (Chen et al. 2018; Fan, Zheng, Lai, et al. 2017; Wang et al. 2006), and these were present in combination with other plan‐based products in many of the other preparations tested.

Oral intake of extracts from all four of these plants have been shown to have anti‐inflammatory effects in animal models of gastrointestinal inflammation (e.g., reduced TNF‐alpha and other cytokines) and, in many cases, to increase the expression of the tight junction proteins (e.g., Zonulin) and reduce intestinal permeability which is often impaired in IBS patients due to sub‐clinical mucosal inflammation. Beneficial effects on the colonic microbiome were also documented (Yan et al. 2022; Zhou et al. 2017; Liu et al. 2021; Wu et al. 2021; Kai et al. 2022).

Based on the information available, TCM appears to be effective in the treatment of IBS patients. Preparations containing the four basic components of Tongxie are the best tested; however, it is unclear which exact mixture is most effective. This limitation, together with the variable quality of the studies performed, reduces the strength of recommendation that can be made (Layer 2021).

### 4.5 Ginger

Ginger has been used for many years in the treatment of functional gastrointestinal symptoms, especially nausea (van Tilburg et al. 2008; Tan et al. 2023). A study by van Tilburg et al. could not show any efficacy of ginger over placebo in IBS patients (van Tilburg et al. 2014); however, Al‐Jassim et al. demonstrate that ginger could be helpful in IBS‐C patients for abdominal bloating and stool regulation (Al‐Jassim 2019). Few adverse events like heartburn were recorded. The Risk of bias assessment showed some concerns in both studies. A meta‐analysis of Ginger could not be performed because the data in one of the two RCTs were only presented as the relative change in IBS‐Severity Scale compared to baseline (Al‐Jassim 2019).

Ginger is of interest due to well described anti‐emetic and spasmolytic effects (Ghayur and Gilani 2005). In addition, clinical studies have shown that ginger has an effect on pain (Terry et al. 2011). It is possible that ginger will find a place in the treatment of IBS; however, at present, the evidence base is insufficient to make any recommendation.

### 4.6 Other Plant‐Based Preparations

Other plant‐based preparations have been tested for effects on IBS patients (24 studies including 2206 patients); however, there was insufficient data for inclusion in the meta‐analysis (Table S1). Eight of these studies applied extracts from single plants and 16 more complex mixtures. All but three studies that met the entry criteria were RCT. Overall, 16 studies were positive and 8 did not demonstrate efficacy in IBS patients. No serious adverse events were reported.

To illustrate the range of products tested, the results of selected positive and negative studies are highlighted.

Aniseed oil was studied in a randomized, double‐blind, parallel‐group clinical trial (*n* = 120) and demonstrated a greater reduction in bloating and abdominal pain in IBS patients compared not only to placebo but also to peppermint oil (dose not stated) (Mosaffa‐Jahromi et al. 2016). The results from this single center study need to be confirmed in further studies.

The effect of STW5 (Iberogast), a mixture of herbal products, in IBS patients was assessed in a multi‐centre, randomized, double‐blind, placebo‐controlled study (*n* = 208) and showed improvement in abdominal symptoms compared to “bitter candytuft” and placebo (Madisch, Holtmann, Plein, and Hotz 2004). The mechanism of action for Iberogast, already used in functional dyspepsia (Madisch, Holtmann, Mayr, et al. 2004; von Arnim et al. 2007; Madisch et al. 2001; Braden et al. 2009; Rösch et al. 2002), includes both antispasmodic and prokinetic effects that appear to improve digestive function (Okpanyi et al. 1993).

The effects of activated charcoal in comparison to nonactivated charcoal were compared in a double‐blind, randomized, multicentre, prospective clinical trial (*n* = 262) (Hübner and Moser 2002). Both substances improved IBS symptoms by up to 60%; however, the lack of a placebo group makes interpretation difficult. In principle, the high absorption capacity of charcoal could be useful in binding excess bile acids and other potential irritants that can cause diarrhea and other symptoms in IBS patients (Hübner and Moser 2002).

The use of plant‐based medications that improve psychological well‐being in IBS patients is attractive because, although certain antidepressants can reduce functional abdominal pain, (Ford et al. 2023) many patients are reluctant to start this form of treatment. It is thought that St. John's wort has antidepressant effects that are mediated by the serotonergic system (Yu 2000); however, a randomized controlled trial (*n* = 70) did not show any benefit compared to placebo (Saito et al. 2010). Increasing evidence indicates that tricyclics are more effective than serotonin reuptake inhibitors in the treatment of chronic abdominal and other pain syndromes (Xie et al. 2015) and this may be the reason why St John's wort lacked efficacy in IBS patients.

### 4.7 Limitations

Strengths of this systematic review and meta‐analysis include the robust search strategy and rigorous methodology that was applied (Bayer et al. 2021). This process was based on guidelines from the Mainz University Library and overseen by an experienced medical librarian (LCO). One limitation was the use of different diagnostic criteria for IBS diagnosis over the period covered by the review. For example, the prevalence of IBS assessed by the current Rome IV classification is approximately half that when Rome III is applied (Palsson et al. 2020). This is primarily because the presence of pain and not, as previously, pain or discomfort is now required to meet the diagnostic criteria. Such changes alter the inclusion and exclusion criteria for clinical trials and could also on the apparent efficacy of treatments.

Further limitations are related to the design of original studies included in the review, including small population size, short study duration (often only 4 weeks), imperfect randomization and blinding procedures, all of which are important to mitigate the high placebo response seen in all disorders of gut‐brain interaction (Patel et al. 2005). The risk of bias assessment identified some concerns in most studies. Additionally, there was minor variation in the composition of some plant‐based products (especially Chinese Herbal Medicine). Furthermore, the efficacy of the plant‐based preparations has only been replicated in a few studies. To counter these factors a strict intention‐to‐treat analysis was carried out for the meta‐analyses; drop‐outs were considered as a failure of therapy, and a random effects model was used so that the therapeutic effect was not overestimated.

## 5 Conclusion

This research demonstrates that plant‐based therapies can be safe and effective in the treatment of IBS symptoms. Provisional clinical recommendations for the use of these preparations are provided in Table 4. Awareness of these products and their mechanism of action is useful for primary care physicians and gastroenterologists to expand the range of treatment options for patients, many of whom prefer to use “natural,” “non‐pharmacological” options. To date, good evidence is available only for peppermint oil, Tongxie TCM, and soluble fiber; however, the systematic review identified a wide range of other plant‐based therapies that are deserving of further enquiry.

**TABLE 4:** Clinical recommendations based on published data: Use of plant‐based preparations for treatment of IBS symptoms.

Plant‐based preparation	Optimal dosage	Duration of treatment	Indication/IBS subtypes	Grade
Preparations supported by results of meta‐analysis				
Peppermint oil Commercial preparations may also contain caraway oil	2–3 × 180 mg/day encapsulated Also used on an “as required “basis	4–8 weeks Preliminary evidence supports prolonged treatment	Treatment of abdominal pain and bloating in IBS‐C, IBS‐D and IBS‐M patients	Low‐moderate
Dietary Fiber, Milled ispaghula husk and other soluble fibers less likely to cause abdominal bloating than insoluble fiber such as bran	2–3 × 10 g (dessert spoon) in 250 ml water/day Regular intake may increase chance of normal stool consistency and frequency	4–12 weeks If required, fiber supplements can be taken over long periods	Treatment of irregular stool consistency and frequency. Most studies recruited IBS‐C and IBS‐M patients	Low‐moderate
Chinese Herbal Medicine Best evidence available for “Tongxie” prep‐arations (see text)	2–3× recommended dose/day	4–8 weeks	Studies report improvement of all IBS symptoms. All published studies in IBS‐D patients	Low‐moderate
Preparations supported by individual studies				
Aniseed oil	200 mg 3× daily encapsulated	4 weeks	Study reports improvement of IBS symptoms superior to peppermint oil	Low
Iberogast (STW5)	20 drops 3× daily	4 weeks	Study reports abdominal pain score and irritable bowel syndrome symptom score were reduced	Low
Activated charcoal	1–8 tablets per day, depending on individual response to therapy	12 weeks	Improvement of irregular stool consistency and frequency. Studies recruited IBS‐D and IBS‐M patients	Low

## Author Contributions

**Anselm Meyer:** conceptualization (equal); data curation (lead); formal analysis (lead); investigation (lead); validation (lead); writing – original draft (lead); writing – review and editing (equal). **Jonas Auer:** conceptualization (supporting); validation (equal); writing – review and editing (equal). **Lorena Cascant‐Ortolano:** validation (equal); methodology (lead); writing – review and editing (equal). **Niclas Schreiber:** data curation (equal); investigation (equal); validation (equal); writing – review and editing (supporting). **Amy Zimmermann‐Klemd:** validation (equal); writing – review and editing (equal). **Carsten Gründemann:** methodology (equal); validation (equal); writing – review and editing (equal). **Philipp Busche:** methodology (equal); validation (equal); writing – review and editing (equal). **Jörg Schirra:** methodology (equal); validation (equal); writing – review and editing (equal). **Mark R. Fox:** conceptualization (lead); data curation (equal); formal analysis (equal); investigation (lead); methodology (lead); supervision (lead); validation (equal); writing – original draft (equal); writing – review and editing (lead). All authors have approved the final draft of the manuscript.

## Conflicts of Interest

The authors declare no conflicts of interest.

## Supporting information


**Data S1:** Supporting Information.

## Data Availability

The data that support the findings of this study are available from the corresponding author upon reasonable request.
