# Supplementary material for: Systematic Review and Meta‐Analysis: Plant‐Based Therapy for Irritable Bowel Syndrome
Source: Phytother Res. 2026 Aug 9;40(10):5918–50. doi: 10.1002/ptr.70421 (PMC13636137; doi:10.1002/ptr.70421)
Supplement: Supplementary file 1 [file PTR-40-5918-s001.docx]

| **Author**  **Year,**  **Country** | **Study-**  **Design and (Duration**  **(weeks))** | **Completed/**  **Sample Size**  **(% female)** | **Mean**  **Age (Ye-**  **ars)** | **Diagnostic-**  **Criteria**  **(%Subtype)** | **Therapy vs. Control**  **(Dose)** | **Endpoints** | **Outcomes** | **Side Effects (Intervention- vs. control group)** |
| --- | --- | --- | --- | --- | --- | --- | --- | --- |
| **Other plant-based preparations** | |  |  |  |  |  |  |  |
| Agah  2015,  Iran | pre- vs. post- treatment studie  (4 weeks) | 45/45 | ran-  ge:  10-50  years | Rome III | Chamomile  (2x10 drops/d) | Change in IBS symptoms | IBS symptoms were reduced at the second and fourth week (p<0.001). |  |
| Alt  2017,  Germany | RCT  (8 weeks) | 86/90  (70%) | 46  years | Rome III | Xanthofen (IQP-CL-101) vs. placebo  (2x330 mg/d) | IBS-Symtom Severity Score | IBS-SSS decreased more strongly with IQP-CL-101 than placebo after 8 weeks (−113 vs −39 points; p<0.001). |  |
| Bordbar 2020,  Iran | RCT  (2 weeks) | 58/64  (61%) | 34  years | Rome III  (23% IBS-C, 36% IBS-D, 41% IBS-M) | Herbal Softgel Capsule (essential oils: Zataria multiflora Boiss, Trachyspermum ammi L. and Anethum graveolens L.) vs. hyoscine butylbromi-de (2x180 mg/d) | IBS Symptom Severity Scale,  IBS Adequate Relief,  IBS Global Assessment Improvement | Posttreatment improvement percentage for IBS-AR, IBS-GAI, and IBS-SSS was higher in the intervention group (83.9%, 75%, and 87%) than in the control group (37.9%, 27.5%, and 34.4%), (P<0:001).” | 2 vs. 10 cases (e.g. abdominal pain) |
| Bortolotti 2011,  Italy | RCT  (6 weeks) | 42/50  (40%) | 45  years | Rome II | Red pepper vs. placebo  (4x150 mg  /d,  in 8 patients reduction to 2x150mg/d) | Abdominal pain and bloating | Abdominal pain and bloating mean score values were significantly improved in the red pepper group but not in the placebo group. | Red pepper group:  14 cases (abdominal pain) |
| Brinkhaus 2005, Germany | RCT  (18 weeks) | 90/106  (63%) | 48  years | Author-defined IBS | Curcuma xanthorriza (n=24)  Fumaria officinalis (n=24)  vs. placebo  (n=58)  (3x20 mg/d Curcuma xanthorriza  3x250 mg/d  Fumaria officinalis) | IBS-related pain and distension | No therapeutic benefit for fumitory or curcuma over placebo. | 1 (Curcuma group) vs. 8 (Fumaria group) vs. 1 (Placebo group) cases (e.g. constipation and distension) |
| Brown 2015,  USA | RCT  (2 weeks) | 13/16  (81%) | 38  years | Rome III  (100% IBS-C) | Quebracho, Conker Tree and Mentha balsamea Willd oil vs. placebo (150 mg Quebracho, 470 mg Conker Tree and 0,2 ml M. balsamea Wild oil) | Change in constipation and bloating | The average constipation (p=0.0034), bloating (p<0.001) and constipation plus bloating scores (p<0.001) significantly improvent in the herbal extracts group but not in the placebo group. |  |
| Cooke 2000,  UK | pre- vs. post- treatment studie  (4 weeks) | 10/11  (45%) | 39  years | Manning  (100% IBS-D) | Arrowroot  (3x10 ml/d) | Improvement in IBS symptoms | Abdominal pain (z = 2.3, P = 0.02) and diarrhoea (z = 2.0, P = 0.05) improved during treatment. | Arrowroot group: 6 cases (e.g. constipation) |
| Derakhs-  hande  2019,  Iran | RCT  (4 weeks) | 84/90  (63%) | 34  years | Rome III  (34% IBS-C, 30% IBS-D, 29% IBS-M) | Achillea wilhelmsii C. Koch vs placebo  (2x250mg  /d) | IBS-symptom severity index, IBS-Quality of life | IBS-Quality of life improved in the Achillea wilhelmsii C. Koch group and in the placebo group, with no significant difference between groups (p>0.05). |  |
| Hatami 2020,  Iran | RCT  (4 weeks) | 80/112  (58%) | 37  years | Rome IV  (21% IBS-C, 16% IBS-D, 63% IBS-M) | Cumin Sofouf vs.  Mebeverine  (3x 3g sachets/d) | IBS-Symptom Severity Score | “Cumin sofouf was significantly more effective than mebeverine.” | 12 vs. 3 cases (e.g. headache and vomiting) |
| Hawrelak 2010, Australia | two arm, open-label, uncontrol-led pilot study (3 weeks) | 29/31  (84%) | 41  years | Rome II  (32% IBS-C, 68% IBS-D or IBS-M) | IBS-D formulation and IBS-C formulation: (2x1 scoop powder dissolved in 250 ml apple juice/d) | Bowel movements/ day Consistency and frequency of stool Abdominal pain severity | Bowel habit improved with the C-IBS formula but not with the DA-IBS formula in IBS-D or -M. A number of IBS symptoms improved with both formulas. | IBS-D group:  2 cases (e.g.  constipation) |
| Heydari  2023,  Iran | RCT (4 weeks) | 76/101  (55%) | 37  years | Rome III  (100% IBS-D) | Dracoce-  phalum kotschyi Boiss. vs. placebo (3x75 mg/d) | IBS-Severity scoring system  IBS-Quality of life. Tempera-ment Checklist | Quality of life, temperament, and IBS  symptoms improved during treatment. |  |
| Hübner 2002,  Austria, Belgium, Israel and Morocco | RCT  (12 weeks) | 144/262  (67%) | 42  years | Rome I | activated charchoal (Eucarbon®) vs. non-activated charcoal (carbo ligni) (1-3, 4-6, >6 tablets/d) | overall well-being | Symptom improvement with Eucarbon was 60%, with a 9% higher efficacy compared to carbo ligni. | 7 vs 5 cases (affecting the gastrointestinal tract) |
| Jamaliz-  adeh  2021,  Iran | RCT  (4 weeks) | 134/150  (64%) | 37  years | Rome IV  (41% IBS-C, 16% IBS-D, 34% IBS-M) | Zataria multiflora Boiss and Trachyspermum copticum (L.) capsules vs. mebeverine or placebo (2 /d) | Improvement in IBS symptoms | Pain, bloating, and reflux decreased significantly more in the ZT group than in the placebo and mebeverine groups after 4 weeks (P <0.05). |  |
| Kazemian 2017,  Iran | RCT  (4 weeks) | 42/60  (36%) | 39  years | Rome III  (23% IBS-C, 6% IBS-D, 70% IBS-M) | Boswellia carterii, Zingiber officinale, and Achillea millefolium vs. placebo  (3 capsules /d) | IBS-Severity scoring system  IBS-Quality of life | Abdominal pain, bloating, depression and anxiety decreased in the herbal medication group, but not statistically significant. | Boswellia carterii, Zingiber officinale, and Achillea millefolium group: 4 cases (e.g. diarrhea) |
| Lauche 2016, Germany | CO  (4 weeks) | 21/32  (59%) | 50  years | Rome III  (100% IBS-D) | Ayurvedic herbs vs. placebo (2x5g powder dissolved in 100 ml water/d) | IBS-Symptom Severity Score | IBS symptom intensity did not differ between the Ayurvedic herbs group and the placebo group (difference 24.10; 95% CI: -17.12; 65.32, p=0.26). | 9 vs. 4 cases (e.g. diarrhoea, stomach cramps and skin rash) |
| Madisch 2004, Germany | RCT  (4 weeks) | 203/208  (60%) | 44  years | Author-defined IBS  (31% IBS-C, 27% IBS-D, 41% IBS-M) | STW 5 (n=51),  STW 5 II (n=52) and  bitter candytuft mono- extract (n=53) vs. placebo (n=52)  (3x20 drops/d) | IBS symptoms score Abdominal pain scale | Abdominal pain score and irritable bowel syndrome symptom score were significantly reduced in the STW 5 and STW 5-II group compared to placebo group after 4 weeks. | 1 (STW 5 group) vs. 1 (bitter candytuft group) case  (e.g. constipation) |
| Mangel 2008,  USA | RCT  (12 weeks) | 49-67/245  (74%) | 50  years | Rome II  (100% IBS-D) | Crofelemer  (125 (n=62), 250 (n=59) or 500 mg  (n=62)) vs. placebo | stool consistency  adequate relief | Stool consistency or adequate relief were not significantly improved in the Crofelemer group compared to the placebo group. | 58 vs. 21cases (e.g. worsening of symptoms) |
| Mosaffa-Jahromi 2016,  Iran | RCT  (4 weeks) | 108/120  (49%) | 34  years | Rome III  (33% IBS-C, 36%IBS-D, 18% IBS-M) | anise oil capsules (n=40) or  peppermint oil capsules (n=40) vs. placebo (n=40)  (3/d) | Improvement in IBS symptoms IBS-Quality of Life | IBS symptoms improved significantly more with AnisEncap than placebo or Colpermin® after the 4-weeks (…) (P < 0.0001). | 0 (Anise oil group) vs. 10 (Peppermint oil group) vs. 1 (Placebo group) cases (e.g. heartburn) |
| Pazhouh 2020,  Iran | RCT  (6 weeks) | 52/70  (71%) | 34  years | Rome IV  (100% IBS-C) | Anti-IBS Sirup vs. placebo  (3x15 ml/d) | IBS Symptom Severity Scale. Bristol Stool Form Scale | IBS-SSS and BSFS scores significantly decreased compared to baseline after 6-week intervention in both groups (P<0.001). Both scores in the treatment group were lower than in the placebo group after the intervention (P=0.041). | Anti-IBS Sirup group: 5 cases (e.g. headache) |
| Portincasa 2016,  Italiy | RCT  (4 weeks) | 116/121  (66%) | 40  years | Rome III  (38% IBS-C, 62% IBS-D) | capsules (curcumin and fennel essential oil) vs. placebo  (2/d) | IBS- symptom severity score IBS- quality of life | IBS-SSS significantly decreased after 30 days of treatment (50.05 ± 28.85% vs 26.12 ± 30.62%, P<0.001). |  |
| Sahib 2013,  Iraq | RCT  (8 weeks) | 40 | ran-  ge:  25-60  years | Rome III | Capsules (Mentha longifolia, Cyperus rotundus and Zingiber officinale) vs. mebeverine  (3/d) | Change in IBS-Symptoms | IBS symptoms improved with the herbal combination, withs results comparable to Mebeverine after 8 weeks of tretmeant. |  |
| Saito 2010,  USA | RCT  (12 weeks) | 60/70  (86%) | 42  years | Rome II  (30% IBS-C, 39% IBS-D, 31% IBS-M) | St John’s wort vs. Placebo (2x450 mg  /d) | self-reported overall bowel symptom score | BSS decreased in the placebo group significantly lower than in the SJW group at 12 weeks (*P*=0.03). | Placebo group:  1 case (heartburn) |
| Sallon 2002,  Israel | RCT  (12 weeks) | 61/80  (73%) | 47  years | Rome I  (100% IBS-C) | Padma Lax  vs. placebo (2 capsules/  d) | Effect on abdominal pain  stool pattern | Constipation, severity of abdominal pain, and its effect on daily activities, incomplete evacuation, abdomi- nal distension and flatus/flatulence improved significantly in the Padma Lax group compared to placebo after 3 months. | 10 vs. 5 cases (e.g. diarrhea) |
| Vejdani 2006,  Iran | RCT  (8 weeks) | 28/32  (44%) | 39  years | Rome II  (38% IBS-C, 31% IBS-D, 31% IBS-M) | Carmint plus Loperamide or psyllium vs. placebo  (3x30 drops/d) | Abdominal pain  overall relief of IBS symptoms | Severity and frequency of abdominal pain/discomfort were significantly lower in the Carmint group than the placebo group (*P*=0.016, *P*=0.001). | 4 vs. 3 cases (e.g. malaise) |
